# Molecular Basis for Oligomeric-DNA Binding and Episome Maintenance by KSHV LANA

**DOI:** 10.1371/journal.ppat.1003672

**Published:** 2013-10-17

**Authors:** John F. Domsic, Horng-Shen Chen, Fang Lu, Ronen Marmorstein, Paul M. Lieberman

**Affiliations:** Gene Expression and Regulation Program, The Wistar Institute, Philadelphia, Pennsylvania, United States of America; University of Southern California Keck School of Medicine, United States of America

## Abstract

LANA is the KSHV-encoded terminal repeat binding protein essential for viral replication and episome maintenance during latency. We have determined the X-ray crystal structure of LANA C-terminal DNA binding domain (LANA_DBD_) to reveal its capacity to form a decameric ring with an exterior DNA binding surface. The dimeric core is structurally similar to EBV EBNA1 with an N-terminal arm that regulates DNA binding and is required for replication function. The oligomeric interface between LANA dimers is dispensable for single site DNA binding, but is required for cooperative DNA binding, replication function, and episome maintenance. We also identify a basic patch opposite of the DNA binding surface that is responsible for the interaction with BRD proteins and contributes to episome maintenance function. The structural features of LANA_DBD_ suggest a novel mechanism of episome maintenance through DNA-binding induced oligomeric assembly.

## Author Summary

Kaposi's sarcoma-associated herpesvirus (KSHV) establishes latent infections that are associated with several cancers including Kaposi's sarcoma, pleural effusion lymphoma, and multicentric Caslteman's disease. One of the major viral proteins required for establishment and maintenance of the latent state is the latency-associated nuclear antigen (LANA). LANA binds to DNA sequences within the terminal repeats (TR) of the viral genome and stimulates both DNA replication and episome maintenance during latency. Here we present the X-ray crystal structure of the DNA binding domain of LANA (LANA_DBD_) and show that it has the capacity to form oligomeric complexes upon DNA binding. We characterize structural features of LANA_DBD_ that are required for oligomerization, DNA binding, and interaction with host cell BET proteins, BRD2 and BRD4, which are important for mediating multiple functions of LANA, including episome maintenance.

## Introduction

Kaposi's sarcoma-associated herpesvirus (KSHV) is a human gammaherpesvirus that was first identified as the etiological agent of Kaposi's sarcoma and is also associated with pleural effusion lymphomas and multicentric Castleman's disease [1]–[3]. KSHV-associated tumors harbor latent viral genomes that persist as multicopy episomes [4], [5] (reviewed in [6], [7]). During latency the genome is circularized at the terminal repeats (TR), which function as an origin of DNA replication and as sites for tethering the episome to the host cell's metaphase chromosomes [8]–[11]. During latency, the viral episome replicates during S phase using host-cell replication machinery and expresses a limited set of viral proteins and non-coding RNAs responsible for viral genome maintenance and host cell survival [12]–[15].

Latency associated nuclear antigen (LANA) is a 130 kDa multifunctional protein required for TR-dependent DNA replication and episome maintenance during latency [5], [7], [16]–[20]. LANA also maintains latency by suppressing transcription and activity of the lytic trigger Rta [21]–[23]. Additionally, LANA interacts with numerous host cell proteins that mediate viral replication, episome maintenance, transcription regulation, and host-cell survival [15], [18], [24]–[33]. LANA binds to TR DNA through a conserved carboxy-terminal DNA binding domain (DBD) [15], [34]–[38]. LANA_DBD_ shares some common features with the functional orthologs Epstein-Barr virus nuclear antigen 1 (EBNA1) and human papillomavirus E2 [39], [40]. Each of these proteins binds to specific semi-palindromic 16–18 bp viral DNA sequences as an obligate dimer [41]–[44]. LANA binds to two adjacent sites in the 800 bp GC-rich terminal repeats, referred to as LANA binding site 1 (LBS1) and LBS2 [42]. Binding to LBS2 is highly cooperative with binding to LBS1 and precisely phased binding to both LBS1/LBS2 is essential for DNA replication function. Episome maintenance requires at least two LBS1/2 binding sites and the viral genome consists of 30–40 terminal repeats [4], [45]. The precise mechanism of DNA binding and how DNA binding and spacing confers replication and episome maintenance remains poorly understood.

There are at least two distinct mechanisms by which LANA can tether viral episomes to metaphase chromosomes. The extreme N-terminus of LANA can interact with host chromosomes through a direct interaction with histones H2A and H2B [46], [47]. A second independent mechanism involves the interaction of the LANA_DBD_ with host chromatin-associated protein [30], [48]–[50]. Prominent among these are the BET proteins BRD2 and BRD4, which contain two bromodomains that bind to the acetylated tails of histones H3 and H4, and a conserved C-terminal extraterminal (ET) domain [51], [52]. The BRD ET domains interact directly with the DBD of LANA, providing a linkage between LANA and acetylated histone tails [53], [54]. In both mechanisms, tethering requires a sequence-specific interaction between the LANA_DBD_ and the viral episome at LBS1/2.

To better understand the mechanism of LANA function through DNA binding we determined the crystal structure of LANA_DBD_. This structure reveals several remarkable features that provide new insight into the regulation and function of LANA. We found that LANA can form a higher-order decameric ring structure. Mutagenesis studies demonstrate that the hydrophobic interface between LANA dimers is important for cooperative DNA binding, DNA replication, and episome maintenance. We also show that an amino terminal arm is important for DNA binding and replication function. Finally, we demonstrate that the BRD ET domain interacts with a basic patch located opposite to the DNA binding face that is crucial for episome maintenance.

## Results

### The crystal structure of LANA_DBD_ reveals a higher-ordered organization

**Figure 1:**
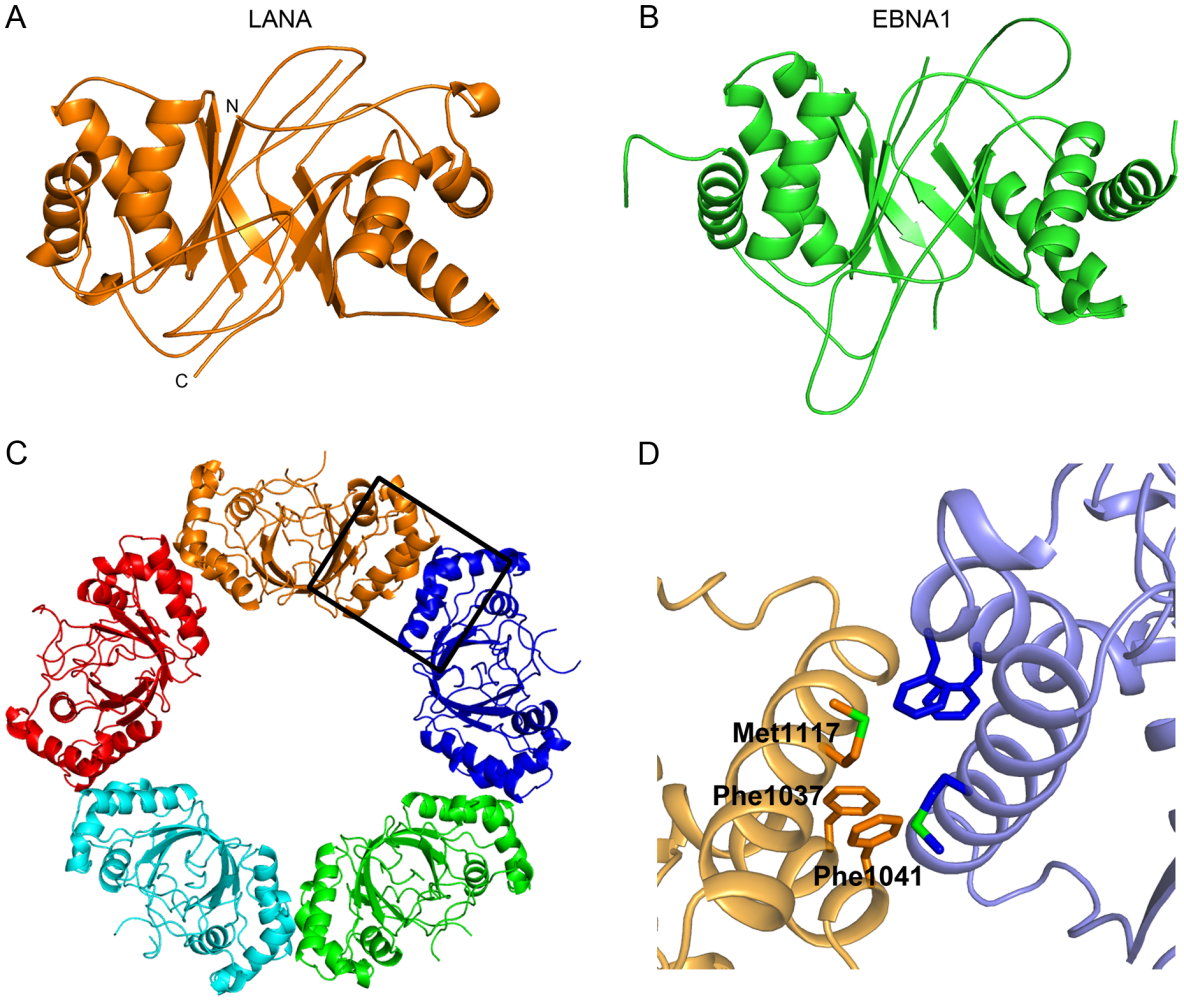
Crystal structure of LANA_DBD_. (A) The structure of the LANA_DBD_ dimer is shown in orange. (B) This fold is conserved in the functional homolog EBNA1 (PDB 1vhi; [55]. (C) In the crystal structure, five LANA_DBD_ dimers interact to form a decameric ring. Each dimer is highlighted in a different color. (D) A zoom-in of the boxed area of (C) highlights the residues involved in the formation of the tetramer interface. It is composed of Phe1037, Phe1041 and Met1117, each shown as sticks.

To characterize the structural properties of LANA_DBD_, we crystallized a construct encompassing residues 1011–1153 and determined its X-ray crystal structure at 2.05 Å resolution. A comparison with the DBD of EBNA1 shows that the two proteins share the same general fold (RMSD of Cα atoms = 5.1 Å) despite sharing very little amino acid sequence homology [55] (Figures 1A, 1B, and S1). A central, anti-parallel beta-sheet forms a hydrophobic core through which two proteins subunits form a dimer. Three helices, which contain the key residues involved in DNA binding, flank this core region [56]. The most notable differences between the homologs are in the intervening loop regions, with LANA_DBD_ exhibiting a more compact structure.

Remarkably, the crystal structure of LANA_DBD_ reveals a higher-ordered assembly comprised of five dimers interacting end-to-end, forming a decameric ring with an exterior diameter of 110 Å and an interior diameter of 50 Å (Figure 1C). The dimers are arranged such that the DNA binding surface faces the exterior of the decameric ring. The interface between the dimers is small and hydrophobic, consisting of residues Phe1037, Phe1041, Met1117, Ala1121, and Ala1124, and buries a total solvent excluded surface area of 963 Å^2^ (Figure 1D).

### The oligomeric interface affects DNA binding and cooperativity

**Figure 2:**
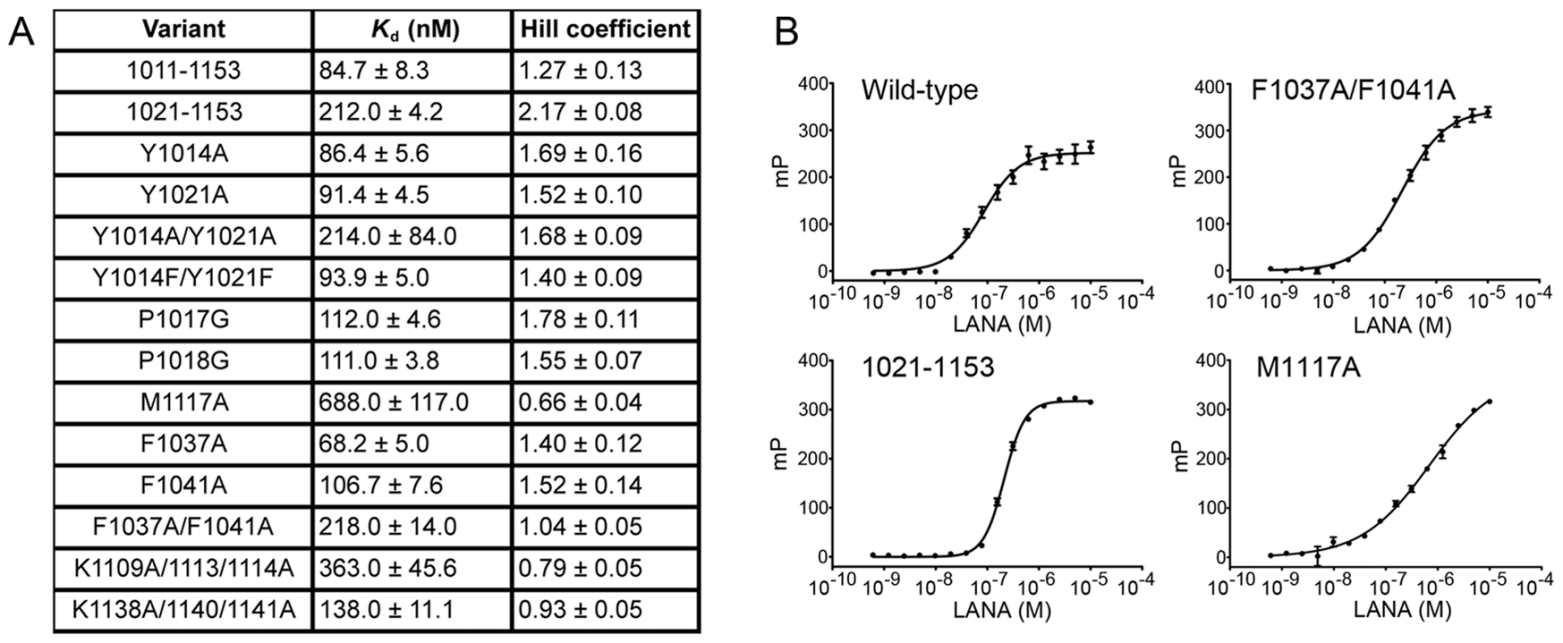
Oligomeric interface affects DNA binding and cooperativity. (A) Dissociation constants for mutants of LANA_DBD_ as determined by fluorescence polarization using an LBS1 oligomer. (B) Representative isotherms of the FP assays for wild-type (cooperative), F1037A/F1041A (non-cooperative), 1021–1153 (increased cooperativity), and M1117A (negative cooperativity) to illustrate the fit of the data using a Hill coefficient. The error bars represent the standard deviation of three independent replicates. Fits not shown in this panel are available in Figure S3.

Cooperative DNA binding has been described for LANA at the KSHV TR LBS1/2 binding sites [42]. To assess the role of the tetramer interface of LANA_DBD_ in cooperative DNA binding, we used a fluorescence polarization (FP) assay with LBS1 and fit the data using a single binding site model with a Hill coefficient (h). We then created LANA mutants F1037A/F1041A and M1117A that lie within the tetramer interface. We found that wild-type LANA_DBD_ exhibits cooperativity in DNA binding (h >1). However, F1037A/F1041A and M1117A had reduced DNA binding affinity, with 38% and 12% of wild-type affinity, respectively (Figure 2). Moreover, the F1037A/F1041A mutation resulted in a complete loss of cooperativity (h = 1). As these residues are not positioned near the DNA binding face, it is likely that the reduced binding affinity observed is due to reduced cooperativity. Since only a single site was used in this assay, there is most likely a structural change that occurs upon DNA binding that primes a second LANA_DBD_ molecule for DNA interaction.

### DNA binding induces oligomerization in vitro

**Figure 3:**
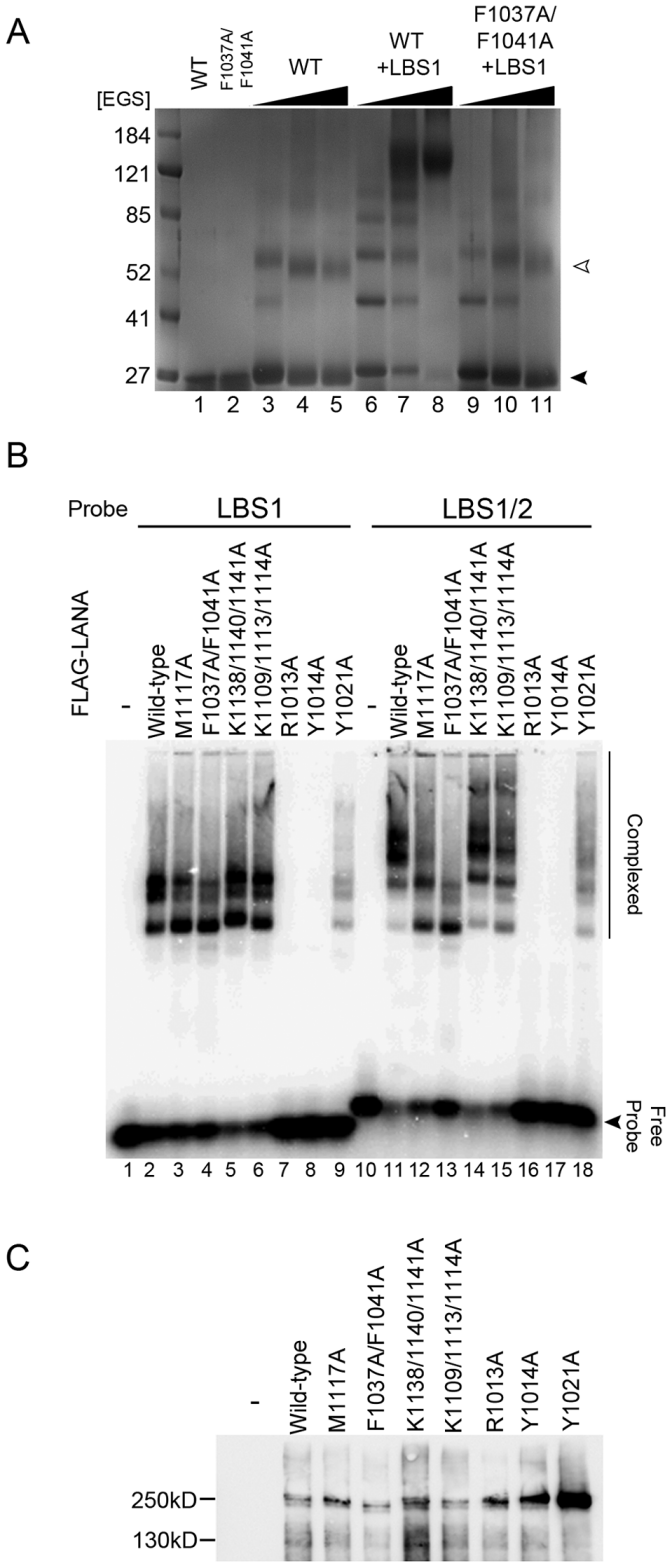
The tetramer interface is required for cooperative DNA binding. (A) DNA binding induces oligomerization of LANA_DBD_. In the absence of crosslinker (EGS), wild-type and F1037A/F1041A migrate as monomers (lanes 1 and 2). Addition of increasing concentrations of EGS produces mostly dimers and tetramers of wild-type (lanes 3–5). Addition of LBS1 DNA induces oligomerization (each successive band is the addition of one monomer) in wild-type (lanes 6–9). The oligomer interface mutant F1037A/F1041A has a greatly reduced propensity to form higher molecular weight oligomers (lanes 9–11). (B) Agarose gel EMSA of full-length FLAG-tagged LANA wild-type or mutant proteins (indicated above each lane) binding to DNA probes for LBS1 (lanes 1–9), or LBS1/2 (lanes 10–18). (C) Western blot of affinity purified FLAG-LANA proteins used for EMSA in panel B.

To determine if the oligomeric states of LANA that are observed in the crystals exist in solution, we performed chemical crosslinking experiments both with and without DNA. In the absence of DNA, we observed the formation of two crosslinked complexes with a molecular weight corresponding to a dimer and a tetramer (Figure 3A). However, when LBS1 DNA was added, a laddering of crosslinked complexes occurred with a maximum molecular weight of ∼150 kDa, corresponding to approximately a decamer. This suggests that DNA binding induces oligomerization of LANA_DBD._

To further investigate the role of the oligomeric interface in DNA binding-induced oligomerization of LANA_DBD_, we assayed LANA mutants by electrophoretic mobility shift assay (EMSA) using full length LANA derived from human cells (Fig. 3B). In the presence of LBS1 DNA, we found that full-length LANA formed multiple oligomeric nucleoprotein complexes, indicative of DNA binding-induced oligomerization (Figure 3B lanes 1–9). When LBS1/2 DNA was used, we observed the formation of tetramer/DNA complexes as well as additional higher-ordered oligomeric species (Figure 3B lanes 10–18). Mutations predicted to disrupt the tetramer interface, M1117A and F1037A/F1041A, were capable of binding LBS1 but showed greatly reduced capacity to form oligomeric species. Furthermore, M1117A exhibited reduced ability to form fully occupied LBS1/2 complexes and F1037A/F1041A was severely impaired in forming higher order LBS1/2 DNA complexes (Figure 3B, lanes 12–13). These results indicate that hydrophobic residues at the tetramer interface are essential for higher order protein-DNA complex formation and cooperative DNA binding.

### The oligomeric interface of LANA_DBD_ is essential for DNA replication and episome maintenance

**Figure 4:**
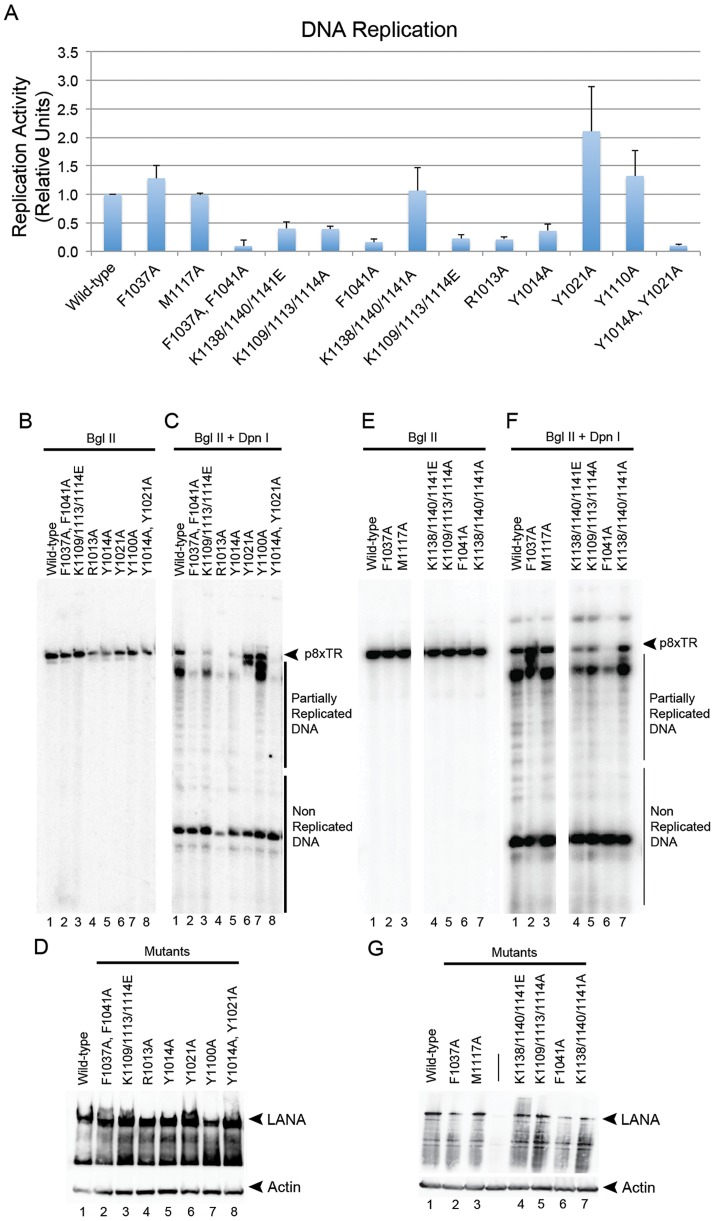
Plasmid replication activity is dependent on DNA binding activity and the oligomerization interface. (A) Quantification of DNA replication assays of wild-type or mutant LANA (as indicated) after transient transfection with p8xTR in 293T cells. Activity is relative to wild-type LANA and error bars represent the standard deviation for three independent replicates. (B–G) Representative Southern blot replication assay showing BglII linearization (B and E) or BglII+DpnI (C and E) digestion of p8xTR. Arrows indicate full-length DpnI resistant replicated plasmid used for quantification in panel A. Smaller DNA fragments represent unreplicated input or incomplete replication products of p8xTR plasmid. (D and G) Western blot for detection of LANA proteins used in replication assays.

**Figure 5:**
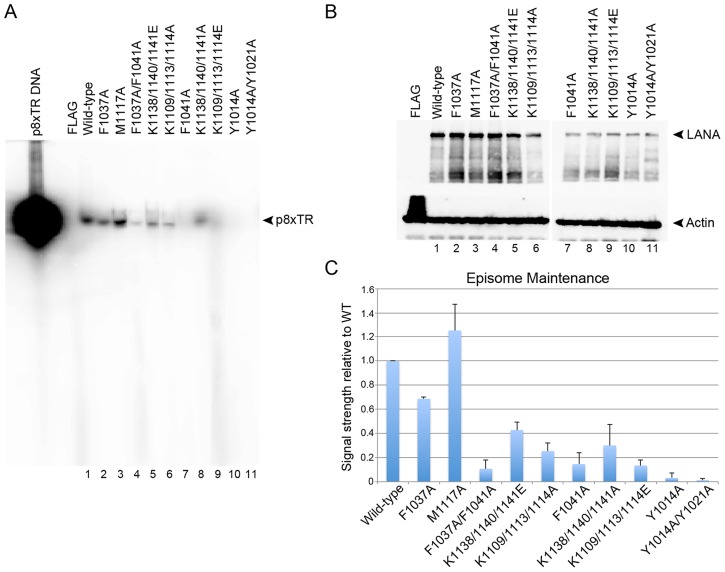
Mutations that affect DNA binding and oligomerization are deleterious to plasmid maintenance activity. (A) Southern blot analysis of p8xTR plasmids at 7 days post-transfection in BJAB cell lines expressing wild-type or mutant LANA, as indicated above. The same number of cells were loaded in each lane. (B) Western blot of LANA protein expression levels in BJAB cells used for plasmid maintenance assays shown in panel A probed for LANA or cellular Actin. (C) Quantification of plasmid maintenance assays normalized to wild-type LANA (lane 1). Error bars represent the standard deviation for three independent replicates.

To determine if mutations that disrupt oligomerization and cooperative DNA binding are important for LANA function *in vivo*, we tested these and additional mutations in the context of full-length LANA using plasmid replication and plasmid maintenance assays (Figures 4 and 5). The plasmid contained eight consecutive terminal repeats (p8xTR) and replication was assessed 72 hours post-transfection by measuring the levels of DpnI resistant plasmid DNA (Figure 4). Mutations that disrupted the oligomerization interface (F1037A/F1041A) completely abrogated DNA replication function (Figure 4). All of these proteins were expressed at similar levels as measured by Western blot analysis (Figure 4D and G).

LANA mutations were also assessed for their ability to support long-term plasmid maintenance in B-lymphoid cell lines (Figure 5). Full length LANA and LANA mutant proteins were selected for stable expression in BJAB B cell-lymphoma cell lines, and then co-selected with plasmids containing p8xTR and a G418-resistance marker. While most LANA mutations were expressed at similar protein levels to wild-type LANA, their ability to support plasmid maintenance varied substantially. Single substitution mutations F1037A and M1117A supported nearly wild-type levels of plasmid replication. In contrast, the F1041A mutant showed impaired replication and maintenance activity and the double mutant F1037A/F1041A, which disrupts oligomeric interactions, was nearly completely void of episome maintenance function.

### The N-terminal arm (NTA) of LANA_DBD_ is important for DNA binding

**Figure 6:**
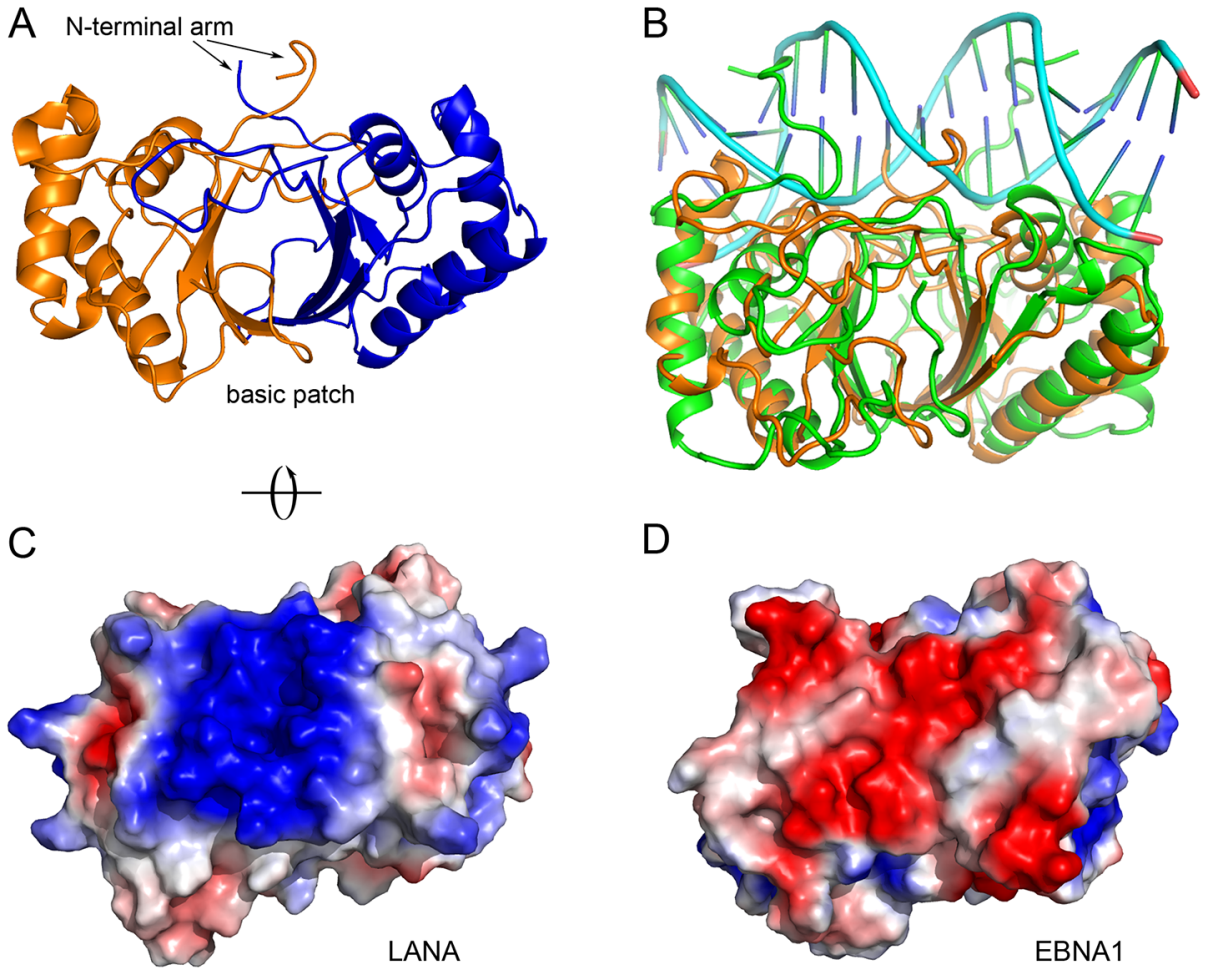
LANA_DBD_ presents unique surface features. (A) The N-terminal arm of the domain lies across the DNA binding face. (B) Superimposition with the structure of EBNA1 bound to DNA (PDB 1b3t; [59]) shows that, in this position, the NTA of LANA_DBD_ would occlude DNA from binding. (C) An electrostatic surface potential rendering (red = acidic, blue = basic and white = neutral) highlights the coverage of this basic patch opposite the DNA binding face on LANA_DBD_. This view is rotated 90° about the x-axis in (A), as indicated. (D) In EBNA1, the same patch is generally acidic, with a slightly lower overall charge density.

In the crystal structure of EBNA1_DBD_ bound to DNA, the N-terminus of the domain acts as an arm that wraps around the minor groove of the DNA. In our DNA-free structure of LANA_DBD_ the N-terminal portion of this construct lies across the DNA binding face, based on homology with the EBNA1 co-crystal structure (Figure 6A and 6B). We anticipate that, when bound to DNA, this arm undergoes a conformational change to allow DNA recognition with the conserved DNA contact residues, and wrap around the DNA similarly to that observed in EBNA1_DBD_. To determine if this arm plays a role in DNA binding we prepared several mutants, R1013A, Y1014A, P1017G, P1018G, Y1021A, Y10141A/Y1021A, and Y1014F/Y1021F, and an N-terminal deletion construct 1021–1153.

As measured by FP and EMSA, mutation of P1017 or P1018 to glycine caused a slight decrease in affinity to about 75% compared to wild-type (Figure 2). In FP assays, Tyr1014 and Tyr1021 could be singly mutated to alanine with no detriment to DNA binding affinity. Furthermore, the Y1014F/Y1021F double mutant had nearly the same binding affinity as wild-type. However, the Y1014A/Y1021A double mutant had approximately 40% binding affinity compared to wild-type. This effect was comparable to the N-terminal deletion construct 1021–1153. Interestingly, single alanine substitutions of Tyr1014 and Arg1013 completely disrupted DNA binding in EMSA (Fig. 3). Although the differences between FP and EMSA are several, including different protein sources and different biophysical parameters, the results substantiate the importance of the N-terminal arm in stabilizing LANA-DNA binding.

Generally, the results of the *in vivo* assays agreed with the FP data. Mutants R1013A, Y1014A, and Y1014A/Y1021A showed greatly reduced levels of replicated plasmid with Y1021A also showing some reduction (Figure 4). These same mutants also had a reduced capacity to support the persistence of p8xTR in plasmid maintenance assays (Figure 5). Based on this data, we conclude that the NTA of LANA_DBD_ participates in DNA binding and is required for replication and episome maintenance function *in vivo*.

### A non-DNA interacting basic patch of LANA interacts with the ET domain of BRD proteins

The surface opposite to the DNA binding face of LANA_DBD_ is a dense basic patch composed mostly of lysine residues (Figure 6C and 6D). This feature appears to be unique to LANA, as the analogous part of EBNA1 is composed mainly of acidic residues (Figure 6D). Previous studies have shown that a region of LANA_DBD_ encompassing a portion of this patch is important for interacting with the ET domain of BRD2 and BRD4 [53], [54].

To further identify which residues are important for this interaction we made mutations of these lysine residues by subdividing the basic patch into two regions, Lys1138/1140/1141, near the dimer interface, and Lys1109/1113/1114, near the tetramer interface. Mutation to alanines resulted in decreased plasmid replication and maintenance functions with K1109/1113/1114A being most impaired (Figures 4 and 5). Further reduction of these activities was achieved by charge reversal mutations to glutamate.

**Figure 7:**
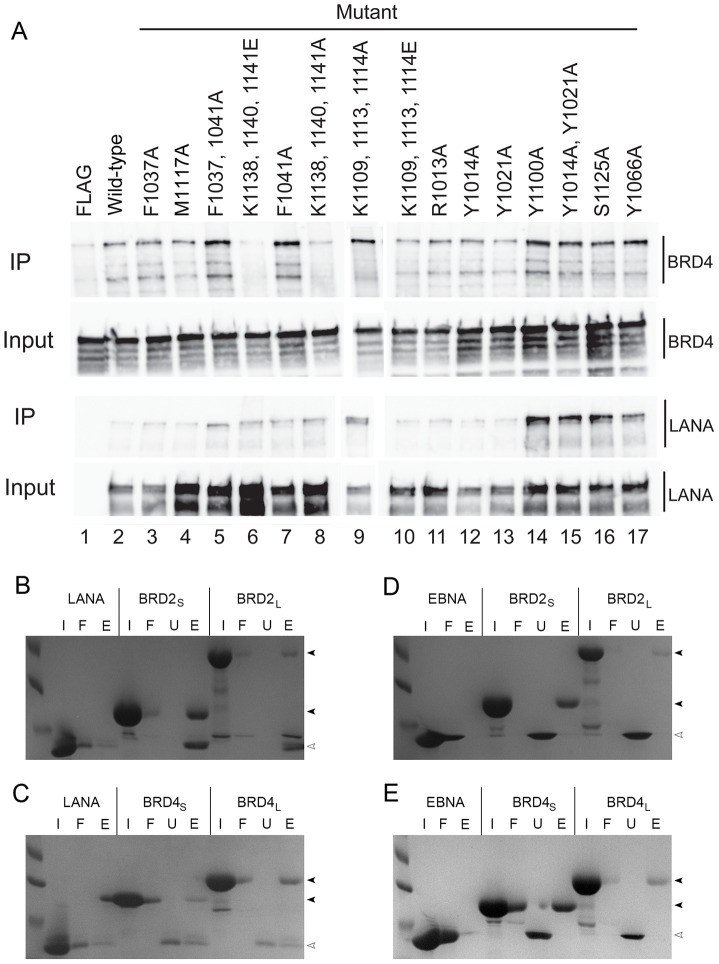
The basic patch of LANA_DBD_ interacts with the ET domain of BRD2 and BRD4. (A) Wild-type LANA (lane 2) is able to co-immunoprecipitate BRD4 and mutations in the N-terminal arm (lanes 11–15), the tetrameric interface (lanes 3–5 and 7), and the interior portion of the basic patch (lanes 9–10) do not affect this interaction. However, mutation of Lys1138, Lys1140, and Lys1141 results in decreased levels of BRD4 interaction (lanes 6 and 8). IP and input are shown for BRD4 (top panels) and LANA (lower panels). (B–E) LANA_DBD_ is able to interact with BRD2 (B) and BRD4 (C) ET domains. Ni-NTA resin was loaded with LANA or His-tagged BRD ET domain (input, I) and the flow-through (F) was collected. Untagged LANA was then added and the unbound (U) fraction was collected. Complex formation is indicated by the presence of BRD (dark arrows) and LANA (open arrows) in the elution fraction (E). EBNA1_DBD_ was not able to interact with either BRD2 or BRD4 ET domains (D, E).

To understand the role that these mutations play in interacting with the ET domain of BRD2 and BRD4, we performed *in vivo* co-immunoprecipitation and *in vitro* pulldowns. Using a FLAG-tagged version of wild-type or mutant full-length LANA, we performed co-IPs looking for the presence of BRD4 (Figure 7A). While wild-type LANA_DBD_ showed binding to BRD4, we found that the K1138/1140/1141A and K1138/1140/1141E mutants exhibited decreased levels of BRD4 interaction. The effect of mutations at Lys1109/1113/1114 was minimal. Other mutations in both the N-terminal arm and the tetramer interface did not show any impairment in interactions with BRD4. These findings indicate that amino acid residues within the basic patch near the dimer interface (Lys1138/1140/1141) are principally responsible for BRD interaction.

To confirm that the co-immunoprecipitation was due to a direct interaction between LANA and the ET domain of BRD we performed *in vitro* pulldowns. For these assays we prepared His-SMT3-tagged versions of BRD2 and BRD4 ET domains and untagged forms of LANA_DBD_ and EBNA1_DBD_ (as a negative control). The long constructs (BRD2_L_ and BRD4_L_) comprised the ET domain with the serine-rich C-terminal tail (Figure S2). The short constructs (BRD2_S_ and BRD4_S_) contained the portion of the ET domain exhibiting the highest sequence identity between the two proteins. The results show that LANA_DBD_ is capable of interacting directly with all four BRD constructs (Figure 7B and 7C), whereas EBNA1_DBD_ did not show any binding to these constructs (Figure 7D and 7E). This data demonstrates that LANA_DBD_ directly binds to the BRD ET domain.

## Discussion

LANA is the major viral protein involved in maintaining the latent state of KSHV infection and ensuring that the viral episome is replicated and passaged with cell division. In this capacity, LANA acts to form a site-specific origin of replication and as a molecular tether between the viral episome and the host chromosome. The C-terminal DNA binding domain of LANA (LANA_DBD_) recognizes specific sites in the viral genome and is essential for each of these activities. Here, we determined the X-ray crystal structure of LANA_DBD_ and formally demonstrated that LANA is a structural ortholog of EBV EBNA1 and papillomavirus E2 DNA binding proteins. Importantly, the structure of LANA_DBD_ presented here reveals several unique features that we have verified are essential for cooperative DNA binding, DNA replication, episome maintenance, and direct interaction with BRD proteins.

### Cooperative DNA binding and oligomerization

The cooperative nature of DNA binding suggests that LANA may form a tetramer prior to DNA binding or that DNA binding induces a conformational change in either the protein or the DNA, which facilitates binding to the lower affinity site, LBS2. We have shown using chemical crosslinking that we can capture LANA_DBD_ tetramers in the absence of DNA and that DNA binding induces the formation of high molecular weight oligomers (Figure 3A). This same behavior was observed using full-length LANA, indicating that oligomerization is not an artifact of the truncated protein (Figure 3B) [37]. Furthermore, cooperativity was observed in FP assays that utilized a single site LBS1 probe, and this cooperativity was reduced by mutations in the tetramer interface (Figure 2). This suggests that tetramerization enhances the interaction with lower affinity sites, such as LBS2.

Cooperative binding has also been observed for LANA's functional homolog in EBV, EBNA1 [57]. EBNA1 binding sites exist in two locations in the latent origin of replication (*oriP*), the family of repeats (FR) and the dyad symmetry (DS) element. Similar to LBS1/2, DS is composed of two sets of tandem EBNA1 binding sites, positioned 21 bp apart, center-to-center [58]. The available structures of EBNA1_DBD_ present a dimeric structure either alone or in the presence of DNA [55], [59]. The residues at the location of the anticipated tetramer interface of EBNA1_DBD_ are generally acidic and, would be unable to participate in a homotypic interaction similar to that seen in LANA. Thus it is likely that a structural change, post-translational modification, or additional cellular protein would be required for the cooperative tetrameric DNA binding observed with EBNA1.

In the crystal structure presented here, LANA_DBD_ dimers interact to form a novel decameric ring structure (Figure 1C). While we have not validated that a decamer forms *in vivo*, the molecular details of the interactions between dimers and the geometric organization of the dimers provide insight into the means by which cooperative binding occurs. The tetramer interface is relatively small and composed of hydrophobic residues Phe1037, Phe1041, Met1117, Ala1021, and Ala1024. We and others have shown that mutation of these residues has adverse effects on DNA binding activity [56]. In particular, the F1037A/F1041A mutant fails to produce DNA binding induced oligomers in solution (Figures 2 and 3). This mutant also fails to form the higher molecular weight complexes with LBS1/2 that are characteristic of full-length wild-type LANA. Most notably, F1037A/F1041A is severely impaired for plasmid replication and maintenance activity (Figures 4 and 5), indicating the importance of the tetramer interface in cooperative DNA binding and LANA functionality *in vivo*.

The geometric arrangement of the dimers in the crystal structure may indicate the manner in which two dimers interact when bound to LBS1/2. Wong and Wilson demonstrated that the binding of LANA to LBS1 induces a 57° bend in the DNA and that binding to LBS1/2 additively increases the bend angle to 110° [60]. Consistent with this, the angle between dimers in the decameric ring is approximately 110° (Figure 1C). Mutational analysis of residues near the tetramer interface located distal to the DNA binding surface further demonstrates that the angle between dimers is important in mediating cooperativity. Met1117 may act to maintain the angle between dimers at approximately 110°. We observed a decrease in the level of oligomeric species formation by the M1117A mutant in EMSA analysis using an LBS1/2 probe (Fig. 3). Additionally, in FP assays (Fig. 2) the *K*_d_ of DNA binding is reduced to about 10% and the Hill coefficient is reduced to less than 1 for M1117A, indicating negative cooperativity. This implies that the angle between dimers in the preexisting tetramers of these mutants exceeds the optimal angle for cooperative binding and the formation of higher ordered complexes. In the context of full-length LANA this geometry may be maintained by additional regions not determined in our crystal structure, as the effects of M1117A are less pronounced in EMSA, replication, and maintenance assays (Fig. 3B, 4, and 5). Taken together, our data supports the role of the tetramer interface as the basis for cooperative DNA binding to TR and functionality in KSHV DNA replication and episome maintenance.

Several other studies support the role of LANA oligomerization in KSHV biology. LANA has been shown to bind TR DNA as an oligomer in EMSA and mutations that disrupt oligomerization were found to block DNA replication and episome maintenance [37]. The oligomerization domain was mapped to the N-terminal arm, which we have also found is required for cooperative DNA binding. Some evidence for oligomeric binding *in vivo* may be inferred from nucleosome mapping studies of the TR in latently infected BCBL1 cells [17]. This study revealed that four nucleosomes are positioned at regular intervals within the 809 bp TR, and a nucleosome-free region of ∼350 bp surrounds the LBS1/2 binding site [17]. This nucleosome-free region could accommodate two LANA dimers and replication factors, and this extended region is essential for efficient DNA replication [38]. It is also possible that one or two LANA decamers could occupy this nucleosome free region, assuming that the each decamer wrapped ∼120 bp of TR DNA. In this model, only one or two dimers of the decamer would have high-affinity interactions with LBS1 and LBS2, and the remaining three dimers would interact non-specifically with adjacent TR DNA. Whether LANA oligomers mediate additional long-distance interactions between tandem TRs with ∼800 bp of intervening DNA is not yet known. However, long-distance interactions have been described for EBNA1, which can form DNA loops between the family of repeats and the dyad symmetry elements of OriP [61]. Higher order oligomeric conformations have also been described for other viral origin binding proteins, including SV40 T antigen and papillomavirus E1, which undergo conformational changes after DNA and ATP-binding [62], [63]. More recently, a complex oligomeric structure has been described for the adenovirus-encoded E4-ORF3, which forms an intracellular network that is important for compartmentalization during viral DNA replication [64]. Interestingly, the structure of E4-ORF3 was shown to share a similar fold to EBNA1, suggesting that LANA, EBNA1, E2, and E4-ORF3 may share some similarities in the self-assembly of larger structures. Thus, it is possible that higher-order oligomerization of LANA plays an important functional role in KSHV biology.

### Flexible N-terminal arm

Another interesting feature of the crystal structure of LANA_DBD_ is the N-terminal arm (NTA) of this domain. Mutagenesis studies revealed that the LANA_DBD_ NTA is essential for stable DNA binding, replication, and episome maintenance function. In the LANA_DBD_ crystal structure, the NTA occupies the exact position where DNA would be located based on superposition with the EBNA1 co-crystal structure (Figure 6B). In the EBNA1_DBD_-DNA co-crystal structure, the N-terminal arm of the domain wraps around the minor groove of the DNA (Figure 6B) [59]. We have shown that mutation of residues located within LANA NTA (specifically Arg1013, Tyr1014, and Tyr1021) decrease DNA binding affinity and causes a loss of plasmid replication and maintenance. It is possible that the NTA occupies this position due to the effects of crystal packing or because the body of the protein presents an entropically favorable environment. However, in this position the arm would occlude DNA from interacting with LANA (Figure 6B). Therefore, it is most likely that the NTA undergoes a conformational change prior to DNA binding, wrapping around the minor groove in a manner similar to that observed in EBNA1. This would also help to explain some of the cooperative oligomerization induced by a single LBS1 DNA biding site. We suggest that DNA binding induces a change in the conformation of the NTA that facilitates higher order oligomerization and cooperative DNA binding by LANA.

### BRD interaction with LANA

A novel feature of LANA_DBD_ observed in the crystal structure is the presence of a lysine-rich basic patch located opposite to the DNA binding. The only prior evidence for a function of the residues within this patch was the observation that deletion of the last 23 amino acids of LANA abrogates interaction with BRD2 and BRD4. These same studies showed that the ET domain of the BRD proteins mediates the interaction with LANA. The sequence of the ET domains of BRD2 and BRD4 reveals two regions that may be important, the N-terminal portion contains a large number of glutamates and the C-terminal tail is serine-rich. By dividing the basic patch on LANA_DBD_ into two parts we were able to show that the internal portion of the patch (Lys1138, Lys1140, and Lys1141) contains the key residues involved in BRD binding (Figure 7A). These lysines appear to interact with the acidic residues in the N-terminal portion of the ET domains (Figures 7B and 7C). We did not observe any interaction with either BRD ET domain with EBNA1_DBD_, as would be expected since the analogous surface of EBNA1 is mostly acidic (Figure 6D).

BRD interactions have been shown previously to mediate LANA function in chromosome binding [53], [54]. Our data is consistent with a role of BRD binding in episome maintenance and DNA replication. However, BRD proteins have also been implicated in other LANA functions, including transcription regulation and cell cycle control. BRD proteins have been shown to mediate multiple functions of E2 family members, including metaphase chromosome tethering, transcriptional repression, and DNA replication. Thus, it is possible that BRDs contribute to multiple functions of LANA.

### Conclusion

The structure and associated biochemical and cell-based studies of LANA_DBD_ reported here reveal new insights into the higher-order oligomerization, cooperative DNA binding, DNA replication, episome maintenance, and BRD interaction interface of LANA. We identified the N-terminal arm, which is crucial for DNA interaction and, based on homology to EBNA1, likely wraps around the minor groove of the cognate DNA, providing for high affinity binding. We have demonstrated that LANA has the capacity to oligomerize upon binding its cognate DNA. The decameric ring observed in the crystal structure provides one possibility for the arrangement of oligomeric LANA, however it is not known for certain if this is the state of oligomerization *in vivo*. We also determined that a basic patch located opposite to the DNA binding face of LANA_DBD_ serves as the interaction site with host cell BRD proteins. These activities are critical for LANA's function in viral DNA replication and tethering the KSHV episome to host cell chromosomes to allow for passage of the genome upon cell division during latent viral infection.

## Methods

### Expression, purification and crystallization

A construct comprising residues 1011–1153 of LANA (LANA_DBD_) was expressed as a His-SMT3 tagged protein in *E. coli*. Cells were sonicated in 2 M NaCl, 25 mM HEPES, 25 mM imidazole, 5 mM beta-mercaptoethanol (BME), pH 8.0 and purified over Ni-NTA resin. The His-SMT3 tag was removed by overnight cleavage with ULP1 protease and the cleaved product was further purified by a second run over Ni-NTA. The protein was then run on a Superdex 75 column equilibrated with 2 M NaCl, 20 mM HEPES, 0.5 mM TCEP, pH 7.4. Mutants were prepared using site-directed mutagenesis and purified as described. The protein was concentrated to 30 mg/mL and crystallization screening was performed. Initial crystallization hits did not provide diffraction beyond 4 Å. To improve crystal quality, crystals grown in 20% polyethylene glycol monomethyl ether 2000, 100 mM Tris pH 8.0, 150 mM calcium acetate were crushed, diluted 1∶10000 in reservoir solution, and used to re-seed entire crystallization screens. This yielded crystals in 1 M ammonium formate and 100 mM HEPES pH 8.0 that diffracted to 2 Å and formed in the monoclinic space group *P*2_1_ (*α* = 51.4 Å, *β = *175.2 Å, *γ* = 97.1 Å, b = 95.3°).

### Phasing and structure refinement

Initial phasing was unsuccessful using molecular replacement with the crystal structure of EBNA1_DBD_ (PDB 1vhi). Crystals were then soaked with a variety of heavy atom salts and potassium osmate was identified as a successful derivatization. Diffraction data were collected to 3 Å at beamline X29a at the National Synchrotron Light Source using wavelengths at the peak (1.1401 Å) and inflection (1.1404 Å). Ten initial osmium sites were located using *AutoSol* in *Phenix*. The phases obtained were of adequate quality to generate electron density maps in which the secondary structure elements could be modeled manually. Once the majority of the protein was built, molecular replacement was performed using a higher resolution native dataset. Model building and refinement were completed using *Phenix*. The model was refined to convergence with R_work_ = 18.18% and R_free_ = 22.62%. Complete data reduction and refinement statistics are given in Table 1. The final structure has been submitted to the Protein Data Bank with accession code 4J2K.

**Table 1:** Crystallographic data reduction and model refinement statistics.

**Crystal parameters**	
Space group	*P* 2_1_
Unit cell	a = 51.439 Å, b = 175.176 Å, c = 97.065 Å, β = 95.3°
**Data Collection**	
Resolution	39.9-2.05 Å (2.12-2.05)*
Unique reflections	101735 (9604)
Redundancy	3.4 (3.3)
Completeness	95.32% (90.60%)
R_merge_	6.7% (53.0%)
I/σ(I)	6.04 (1.77)
**Refinement statistics**	
R_work_	18.18% (28.74%)
R_free_	22.62% (33.17%)
No. atoms (protein/solvent)	10996/553
r.m.s.d.	
Bonds	0.014 Å
Angles	1.39°
Average B-factor	
Protein	47.5 Å^2^
Solvent	45.8 Å^2^
Ramachandran Statistics	
Favored	97.0%
Allowed	3.0%

* Values in parentheses are for highest resolution bin.

### Fluorescence polarization

To determine dissociation constants for DNA binding, fluorescence polarization assays were performed. Protein was serially diluted 2-fold starting at 100 µM in 1 M NaCl, 20 mM HEPES, 1 mM DTT, pH 7.4. These protein samples were then diluted 10-fold into reactions resulting in a final condition of 200 mM NaCl, 20 mM HEPES, 1 mM DTT, 1 µg/mL BSA, 0.1 µg/mL poly(dA:dT) (*Invivogen*, San Diego, CA), 0.001% Tween 20, and 1 nM LBS1 probe (*Integrated DNA Technologies*, Cedar Rapids, IA). This probe was a 21-mer (*AGCGGCCCCATGCCCGGGCGG*) centered on the LBS1 site and was 5′ labeled with 6-carboxyfluorescein. The reactions were incubated at room temperature for 30 minutes and then read using a Beacon 2000 Fluorescence Polarization reader. Data were collected in triplicate and analyzed using a one-site specific binding with Hill slope model in GraphPad *Prism* (version 5.0a; GraphPad Software, La Jolla, CA).

### Protein crosslinking

Crosslinking reactions were performed using ethylene glycol bis[succinimidyl succinate] (EGS; *Thermo Scientific*). The DNA was an unlabeled version of the probe used in the FP experiments. Protein was incubated with a 1.2 molar excess of DNA, where applicable, in a reaction buffer of 200 mM NaCl, 20 mM HEPES pH 7.4, and 1 mM DTT for 30 minutes at room temperature. EGS was dissolved and diluted in DMSO and added to the reaction at 5% of the reaction volume. This was incubated at room temperature and then quenched with 5% volume of 1 M Tris, pH 7.5 for 15 minutes. Samples were boiled with loading dye before loading into gels.

### Electrophoretic mobility shift assays

FLAG-LANA proteins were expressed by transient transfection in 293T cells, and isolated by FLAG-affinity purification after multiple wash steps with extraction buffer (20 mM Tris, 400 mM NaCl, 10% glycerol, 0.5 mM EDTA, 0.05% NP-40, 0.5 mM DTT, and protease inhibitors (Sigma)) to remove weakly associated cellular proteins and nucleic acids. Binding reactions and gel conditions were described previously [65] DNA binding reactions were resolved on a horizontal 1.5% agarose gel in 0.5× TBE (45 mM Tris-borate, 1 mM EDTA) at 10 V/cm for 2 hours and then dried on DE80 paper prior to PhosphorImager exposure. Binding reactions were in a final of 10 µL containing 10 mM HEPES (pH7.9), 10% glycerol, 100 mM KCl, 5 mM MgCl_2_, 0.1 U/µl poly-dIdC, 5 mM β-mercaptoethanol with LANA protein at ∼200 nM and radiolabelled LBS1 or LBS1/2 probe at 2.5 nM.

### Cell culture and transfection

BJAB (uninfected B cell lymphoma) cells or BJAB cells stably expressing triplicate FLAG-epitope-tagged LANA were grown in RPMI medium supplemented with 10% fetal bovine serum (FBS) and maintained at concentrations of 0.2 to 0.8×10^6^/mL. Stable LANA expression was maintained with puromycin selection (2 µg/ml) (Sigma). After cotransfection of p8TR plasmids, LANA stable cells were maintained with both puromycin (2 µg/ml) (Sigma) and G418 (600 µg/ml) (Mediatech) selection.

293T cells were maintained in Dulbecco's modified Eagle's medium (DMEM) containing 10% v/v FBS. For transient transfection experiments, transfection of actively growing 293T cells was processed with Lipofectamine reagent (Invitrogen), and cells were harvested 72 hours post transfection.

For creating stable cells, 10×10^6^ of actively growing BJAB cells were resuspended in 450 µl 10% FBS RPMI media without antibiotics. 30 µg DNA of interest were mixed together with cells in a microcentrifuge tube and incubated at RT for 10 min. All transfections were carried out with the Gene Pulser Xcell (Bio-Rad) setting at 0.22 kv and 960 µF as external capacitor. The transfected cells were incubated at RT for 10 min post electroporation, and then maintained as described above.

### Plasmids

Plasmid p8TR contains eight copies of the TR unit cloned into pREP9 (Invitrogen) (gift of K. Kaye, Harvard Medical School). KSHV LANA was cloned into p3XFLAG-CMV-24 (Sigma) as described previously. All the LANA mutants created described in this study are based on this plasmid. Human BRD4 expression construct was a gift from Dr. Jianxin You (University of Pennsylvania, School of Medicine).

### Immunoprecipitation and protein detection

For immunoprecipitation (IP), co-transfected 293T cells expressing FLAG-tagged LANA wild-type, mutant, or vector only control and human BRD4 expression vector were lysed in 300 µL of IP buffer (50 mM Tris pH 7.6, 60 mM NaCl, 1% glycerol, 0.5 mM EDTA, 0.2% NP- 40, and protease inhibitor (Sigma)) at 4°C with sonication. FLAG-tagged proteins were precipitated with anti-FLAG rabbit serum (Sigma) followed by protein A/G bead (Thermo Scientific) capture.

For immunoblot assays, proteins were resolved in 8–16% Novex Tris-Glycine gels, LANA was detected using an HRP conjugated anti-FLAG antibody (Sigma), and BRD4 was detected using Anti-BRD4 antibody (Bethyl Laboratories, Inc.) at 1∶2000 dilution in conjunction with HRP-conjugated secondary antibodies (GE Life Sciences) and ECL reagents (Invitrogen).

### Hirt DNA extraction

At day 7 post-transfection and selection, BJAB cells were lysed in 1 mL lysis buffer (0.6% SDS, 10 mM EDTA, 10 mM, Tris-HCl pH 7.5, 50 µg/ml RnaseA) per 5×10^6^ cells and incubated at 37°C for 2 hours. NaCl was then added to 1 M final concentration and incubated overnight at 4°C. After a 30 minute centrifugation at 8,500 rpm at 4°C, DNA was extracted once with phenol∶chloform (1∶1), twice with chloroform∶isoamyl alcohol (24∶1), ethanol precipitated, and the DNA pellet was washed with 70% ethanol, air-dried and resuspended in TE buffer.

### Southern analysis

FLAG- or LANA-expressing BJAB cells were transfected with p8TR DNA. After 48 h, cells were maintained in medium containing G418 (600 µg/ml) (Mediatech). Hirt DNA extraction was performed about 40 days post transfection. 30 µg DNA was digested with BglII to linearize the p8TR DNA. Double digestion of 30 µg of Hirt DNA with BglII and DpnI was also performed and electrophoresis in a 0.8% agarose gel in Tris-borate-EDTA buffer. DNA was then transferred to a nylon membrane. KSHV DNA was detected with a ^32^P-labeled TR probes. Quantitation of the linearized BglII- or BglII/DpnI-digested p8TR was performed using a PhosphorImager SI (Molecular Dynamics).

### LANA-BRD pulldowns

BRD2 and BRD4 were expressed in two forms, both as His-SMT3 tagged proteins. The long form encompassed the extraterminal domain including the serine-rich C-terminus (BRD2 633–801, BRD4 601–722). The short form included the portion of the ET domain that is most conserved between BRD2 and BRD4 (BRD2 633–714, BRD4 601–681). The tagged BRD proteins were incubated with Ni-NTA resin (Thermo Scientific) equilibrated with 200 mM NaCl, 20 mM HEPES, 5 mM BME, at pH 8.0 and washed with 10 column volumes (CV) of this buffer. Untagged LANA_DBD_ (1011–1153) or EBNA1_DBD_ (461–642) was then added and the resin was washed again with 10 CV of wash buffer. The protein was then eluted with wash buffer supplemented with 300 mM imidazole. As a negative control the untagged LANA_DBD_ or EBNA1_DBD_ was incubated with the Ni-NTA resin and washed and eluted as described above.

## Supporting Information

Figure S1 **Sequence alignment of the DNA binding domains of LANA and EBNA1.**
(TIF)

Figure S2 **Sequence alignment of the extraterminal domains of BRD2 and BRD4.** Red arrowheads indicate the end of the short constructs.
(TIF)

Figure S3 **Binding isotherms of fluorescence polarization experiments.** The error bars represent the standard deviation of three experiments. The associated *K*_d_ for each mutant can be found in Fig. 2A.
(TIF)

Text S1 **Validation report (rcsb078809) for LANA X-ray crystal structure.**
(PDF)
