# Supplementary figures and images for: Molecular Basis for Oligomeric-DNA Binding and Episome Maintenance by KSHV LANA
Source: PLoS Pathog. 2013 Oct 17;9(10):e1003672. doi: 10.1371/journal.ppat.1003672 (PMC3798644; doi:10.1371/journal.ppat.1003672)

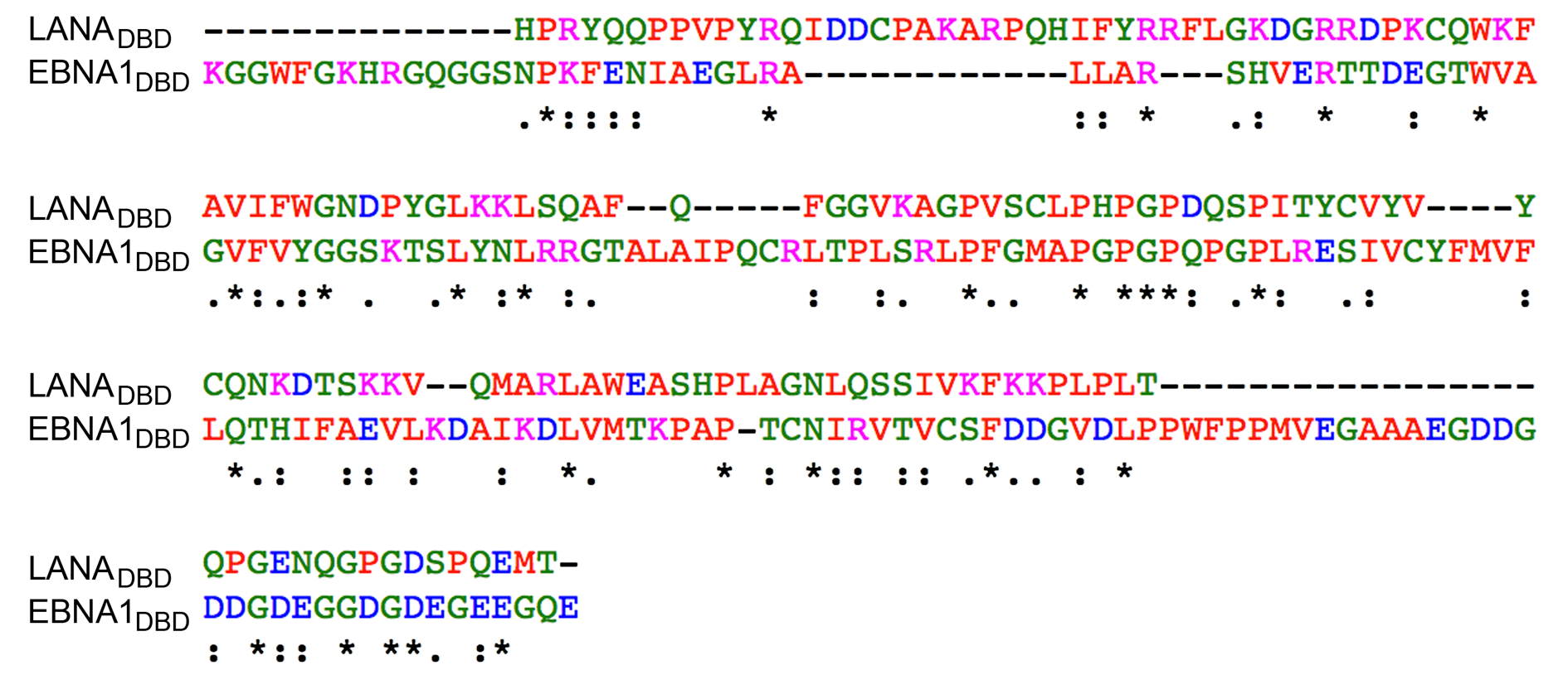

Supplement: Figure S1 — Sequence alignment of the DNA binding domains of LANA and EBNA1. (TIF) [file ppat.1003672.s001.tif]

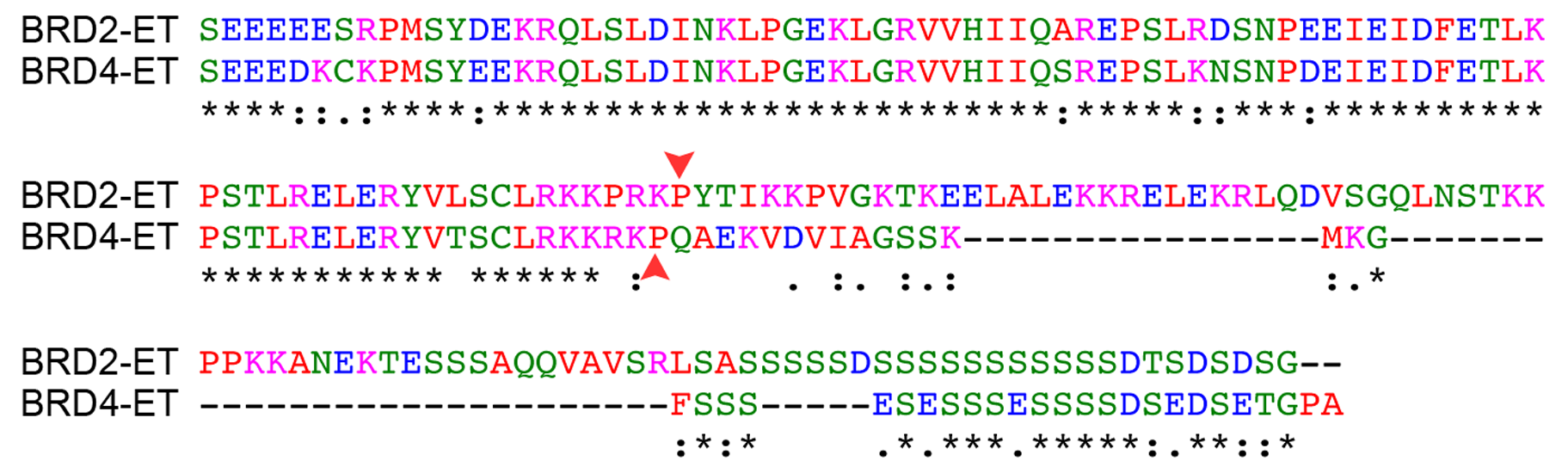

Supplement: Figure S2 — Sequence alignment of the extraterminal domains of BRD2 and BRD4. Red arrowheads indicate the end of the short constructs. (TIF) [file ppat.1003672.s002.tif]

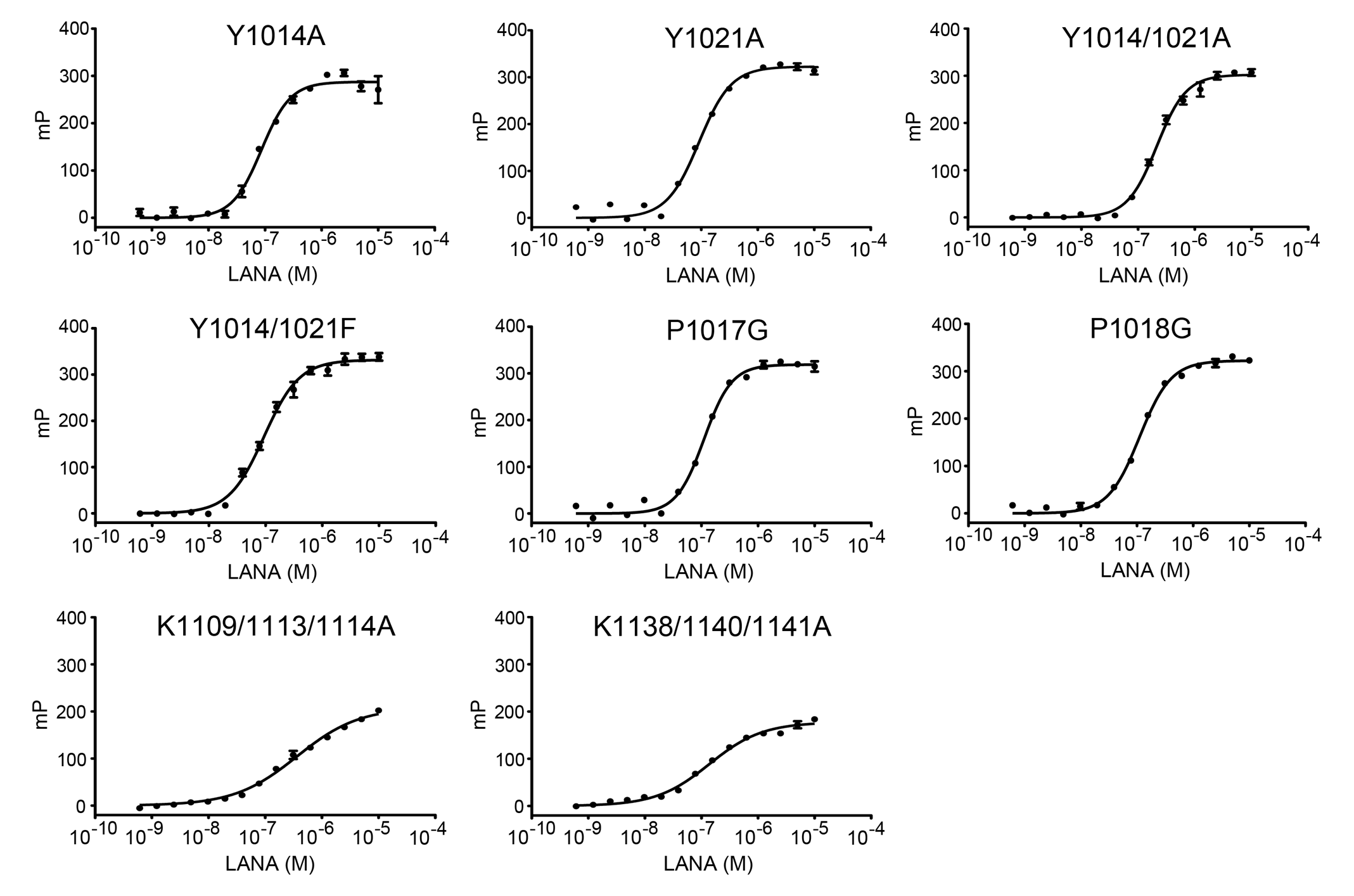

Supplement: Figure S3 — Binding isotherms of fluorescence polarization experiments. The error bars represent the standard deviation of three experiments. The associated K d for each mutant can be found in Fig. 2A. (TIF) [file ppat.1003672.s003.tif]
