# Supplementary material for: Molecular Basis for Oligomeric-DNA Binding and Episome Maintenance by KSHV LANA
Source: PLoS Pathog. 2013 Oct 17;9(10):e1003672. doi: 10.1371/journal.ppat.1003672 (PMC3798644; doi:10.1371/journal.ppat.1003672)
Supplement: Text S1 — Validation report (rcsb078809) for LANA X-ray crystal structure. (PDF) [file ppat.1003672.s004.pdf]

PDB ID : 4K2J
RCSB ID : RCSB078809
TITLE   : Decameric ring structure of KSHV (HHV-8) latency-associated nuclear antigen (LANA) DNA binding domain
AUTHORS : J.F.Domsic, R.Marmorstein

The following geometrical and stereochemical features have been calculated for your structure.

CLOSE CONTACTS

==> Close contacts in same asymmetric unit. Distances smaller than 2.2 Angstroms are considered as close contacts.

Chain	Atom	Res	Seq		Chain	Atom	Res	Seq	Symm_Code	Distance
D	HD2	LYS	1030	-	D	OH	TYR	1066	(1, 5, 5, 5)	Dist = 1.45
C	O2	FMT	1202	-	C	O	HOH	1346	(1, 5, 5, 5)	Dist = 2.02

==> Close contacts based on crystal symmetry. Distances smaller than 2.2 Angstroms are considered as close contacts.

none

BOND DISTANCES AND ANGLES

Bond and angle checks are performed by first computing the average rms error for all bonds and angles relative to standard values for nucleotide units [L. Clowney et al., Geometric Parameters in Nucleic Acids: Nitrogenous Bases, J.Am.Chem.Soc. 1996, 118, 509-518; A. Gelbin et al., Geometric Parameters in Nucleic Acids: Sugar and Phosphate Constituents, J.Am.Chem.Soc. 1996, 118, 519-529] and amino acid units [R.A. Engh and R. Huber, Structure quality and target parameters, International Tables for Crystallography, Volume F, 2001, 382-392]. Any bond or angle which deviates from the dictionary values by more than six times this computed rms error is identified as an outlier.

==> Covalent Bond Lengths:

The overall RMS deviation for covalent bonds relative to the standard dictionary is   0.015 Angstroms

All covalent bonds lie within a 6.0*RMSD range about the standard dictionary values.

==> Covalent Angle Values:

The overall RMS deviation for covalent angles relative to the standard dictionary is   1.4 degrees.

All covalent bond angles lie within a 6.0*RMSD range about the standard dictionary values.

TORSION ANGLES

The torsion angle distributions have been checked. To view these reports, please refer to the ADIT Validation Server at http://deposit.pdb.org/validate.

==> The following table contains a list of torsion angles outside the expected Ramachandran regions [GJ. Kleywegt and TA. Jones, PHI/PSI-chology: Ramachandran Revisited, Structure 1996, 4, 1395 - 1400].

Residue	Chain	Sequence	PSI	PHI
ARG	A	1048	109.60	-57.69
SER	A	1086	-160.20	-120.74
GLN	B	1015	151.34	-42.83
LYS	B	1051	36.98	-91.83
SER	B	1086	-164.74	-127.29
GLN	C	1015	160.43	-45.99
PRO	C	1028	150.57	-45.91
SER	C	1086	-156.03	-127.42
TYR	D	1014	103.86	-53.86
SER	E	1086	-161.68	-127.50
SER	F	1086	-161.98	-128.55
LYS	G	1051	56.35	-92.55
SER	G	1086	-158.39	-130.01
ILE	I	1024	-34.40	-39.01
SER	I	1086	-164.54	-123.17
SER	J	1086	-162.28	-123.63
THR	J	1146	-78.16	-81.12

CHIRALITY

The chirality has been checked. O1P, O2P, and hydrogen atoms which do not follow the convention defined in the IUBMB (Liebecq, C. Compendium of Biochemical Nomenclature and Related Documents, 2nd ed.; Portland Press: London and Chapel Hill, 1992) and IUPAC nomenclature (J.L. Markley, A. Bax, Y. Arata, C.W. Hilbers, R. Kaptein, B.D. Sykes, P.E. Wright and K. Wuthrich, Recommendations for the Presentation of NMR Structures of Proteins and Nucleic Acids, Pure & Appl. Chem., Vol. 70, pp. 117-142, 1998) have been standardized. Any other stereochemical violations are listed below.

none

SOLVENT

The following solvent molecules are further than 3.5 Angstroms away from macromolecule atoms in the asymmetric unit that are available for hydrogen bonding. Solvent molecules in extended hydration shells separated by 3.5 Angstroms or less are not listed.

```
HETATM22117  O   HOH A1342      59.900 144.236   5.356  1.00 54.23      DIST =   3.98 A
HETATM22164  O   HOH B1346      77.563 173.152  15.049  1.00 46.08      DIST =   3.62 A
HETATM22206  O   HOH C1337      74.993 174.033  16.268  1.00 41.60      DIST =   4.50 A
HETATM22319  O   HOH E1370      61.079 210.454 -22.927  1.00 53.15      DIST =   3.92 A
HETATM22321  O   HOH E1372      62.735 206.905 -18.865  1.00 62.45      DIST =   3.64 A
HETATM22384  O   HOH F1356      42.661 231.556 -49.218  1.00 49.17      DIST =   4.08 A
HETATM22393  O   HOH F1365      44.201 237.697 -46.830  1.00 52.12      DIST =   3.99 A
HETATM22419  O   HOH G1325      41.584 232.119 -46.260  1.00 43.18      DIST =   4.60 A
HETATM22449  O   HOH H1325      41.737 202.591 -42.680  1.00 57.38      DIST =   4.61 A
HETATM22457  O   HOH H1333      38.022 209.045 -38.604  1.00 53.89      DIST =   4.69 A
HETATM22459  O   HOH H1335      40.426 200.859 -41.114  1.00 53.57      DIST =   4.29 A
HETATM22517  O   HOH I1348      18.357 159.672 -56.173  1.00 45.98      DIST =   4.53 A
HETATM22559  O   HOH J1337      19.484 157.092 -54.998  1.00 43.51      DIST =   3.77 A
HETATM22570  O   HOH J1348      49.064 147.283 -49.897  1.00 49.92      DIST =   3.68 A
HETATM22572  O   HOH J1350      51.511 154.982 -57.717  1.00 39.34      DIST =   4.50 A
HETATM22576  O   HOH J1354      29.125 145.596 -37.608  1.00 49.85      DIST =   3.59 A
```

We have replaced the coordinates for solvent molecules which could be translated back into the asymmetric unit. Please review all solvent molecules in your file and contact us if you have any serious objections.

MISSING RESIDUES

==> The following residues are missing:
(Note: The SEQ number starts from 1 for each chain according to SEQRES sequence record.)

```
RES MOD#C SEQ

PRO(    A1148 )
```

```
  GLY(    A1149 )
  SER(    B1010 )
  HIS(    B1011 )
  PRO(    B1012 )
  PRO(    B1148 )
  GLY(    B1149 )
  SER(    C1010 )
  HIS(    C1011 )
  PRO(    C1012 )
  PRO(    C1148 )
  GLY(    C1149 )
  SER(    D1010 )
  HIS(    D1011 )
  PRO(    D1012 )
  GLY(    D1149 )
  SER(    E1010 )
  HIS(    E1011 )
  GLY(    E1149 )
  SER(    F1010 )
  HIS(    F1011 )
  PRO(    F1012 )
  SER(    G1010 )
  HIS(    G1011 )
  PRO(    G1012 )
  PRO(    G1148 )
  GLY(    G1149 )
  SER(    H1010 )
  HIS(    H1011 )
  PRO(    H1012 )
  PRO(    H1148 )
  GLY(    H1149 )
  SER(    I1010 )
  HIS(    I1011 )
  PRO(    J1148 )
  GLY(    J1149 )
```

PDB Chain_ID: A

```
        1                                                          15
SEQRES: SER HIS PRO ARG TYR GLN GLN PRO PRO VAL PRO TYR ARG GLN ILE
COORDS: SER HIS PRO ARG TYR GLN GLN PRO PRO VAL PRO TYR ARG GLN ILE
        1010                                                     1024

        16                                                         30
SEQRES: ASP ASP CYS PRO ALA LYS ALA ARG PRO GLN HIS ILE PHE TYR ARG
COORDS: ASP ASP CYS PRO ALA LYS ALA ARG PRO GLN HIS ILE PHE TYR ARG
        1025                                                     1039

        31                                                         45
SEQRES: ARG PHE LEU GLY LYS ASP GLY ARG ARG ASP PRO LYS CYS GLN TRP
COORDS: ARG PHE LEU GLY LYS ASP GLY ARG ARG ASP PRO LYS CYS GLN TRP
        1040                                                     1054

        46                                                         60
SEQRES: LYS PHE ALA VAL ILE PHE TRP GLY ASN ASP PRO TYR GLY LEU LYS
COORDS: LYS PHE ALA VAL ILE PHE TRP GLY ASN ASP PRO TYR GLY LEU LYS
        1055                                                     1069

        61                                                         75
SEQRES: LYS LEU SER GLN ALA PHE GLN PHE GLY GLY VAL LYS ALA GLY PRO
COORDS: LYS LEU SER GLN ALA PHE GLN PHE GLY GLY VAL LYS ALA GLY PRO
        1070                                                     1084

        76                                                         90
SEQRES: VAL SER CYS LEU PRO HIS PRO GLY PRO ASP GLN SER PRO ILE THR
COORDS: VAL SER CYS LEU PRO HIS PRO GLY PRO ASP GLN SER PRO ILE THR
        1085                                                     1099

        91                                                        105
SEQRES: TYR CYS VAL TYR VAL TYR CYS GLN ASN LYS ASP THR SER LYS LYS
COORDS: TYR CYS VAL TYR VAL TYR CYS GLN ASN LYS ASP THR SER LYS LYS
        1100                                                     1114

        106                                                       120
```

```
SEQRES: VAL GLN MET ALA ARG LEU ALA TRP GLU ALA SER HIS PRO LEU ALA
COORDS: VAL GLN MET ALA ARG LEU ALA TRP GLU ALA SER HIS PRO LEU ALA
        1115                                                   1129

        121                                                     135
SEQRES: GLY ASN LEU GLN SER SER ILE VAL LYS PHE LYS LYS PRO LEU PRO
COORDS: GLY ASN LEU GLN SER SER ILE VAL LYS PHE LYS LYS PRO LEU PRO
        1130                                                   1144

        136             140
SEQRES: LEU THR GLN PRO GLY
COORDS: LEU THR GLN   ?   ?
        1145   1147
```

PDB Chain_ID: B

```
        1                                                        15
SEQRES: SER HIS PRO ARG TYR GLN GLN PRO PRO VAL PRO TYR ARG GLN ILE
COORDS:   ?   ?   ? ARG TYR GLN GLN PRO PRO VAL PRO TYR ARG GLN ILE
                    1013                                       1024

        16                                                       30
SEQRES: ASP ASP CYS PRO ALA LYS ALA ARG PRO GLN HIS ILE PHE TYR ARG
COORDS: ASP ASP CYS PRO ALA LYS ALA ARG PRO GLN HIS ILE PHE TYR ARG
        1025                                                   1039

        31                                                       45
SEQRES: ARG PHE LEU GLY LYS ASP GLY ARG ARG ASP PRO LYS CYS GLN TRP
COORDS: ARG PHE LEU GLY LYS ASP GLY ARG ARG ASP PRO LYS CYS GLN TRP
        1040                                                   1054

        46                                                       60
SEQRES: LYS PHE ALA VAL ILE PHE TRP GLY ASN ASP PRO TYR GLY LEU LYS
COORDS: LYS PHE ALA VAL ILE PHE TRP GLY ASN ASP PRO TYR GLY LEU LYS
        1055                                                   1069

        61                                                       75
SEQRES: LYS LEU SER GLN ALA PHE GLN PHE GLY GLY VAL LYS ALA GLY PRO
COORDS: LYS LEU SER GLN ALA PHE GLN PHE GLY GLY VAL LYS ALA GLY PRO
        1070                                                   1084

        76                                                       90
SEQRES: VAL SER CYS LEU PRO HIS PRO GLY PRO ASP GLN SER PRO ILE THR
COORDS: VAL SER CYS LEU PRO HIS PRO GLY PRO ASP GLN SER PRO ILE THR
        1085                                                   1099

        91                                                      105
SEQRES: TYR CYS VAL TYR VAL TYR CYS GLN ASN LYS ASP THR SER LYS LYS
COORDS: TYR CYS VAL TYR VAL TYR CYS GLN ASN LYS ASP THR SER LYS LYS
        1100                                                   1114

        106                                                     120
SEQRES: VAL GLN MET ALA ARG LEU ALA TRP GLU ALA SER HIS PRO LEU ALA
COORDS: VAL GLN MET ALA ARG LEU ALA TRP GLU ALA SER HIS PRO LEU ALA
        1115                                                   1129

        121                                                     135
SEQRES: GLY ASN LEU GLN SER SER ILE VAL LYS PHE LYS LYS PRO LEU PRO
COORDS: GLY ASN LEU GLN SER SER ILE VAL LYS PHE LYS LYS PRO LEU PRO
        1130                                                   1144

        136             140
SEQRES: LEU THR GLN PRO GLY
COORDS: LEU THR GLN   ?   ?
        1145   1147
```

PDB Chain_ID: C

```
        1                                                        15
SEQRES: SER HIS PRO ARG TYR GLN GLN PRO PRO VAL PRO TYR ARG GLN ILE
COORDS:   ?   ?   ? ARG TYR GLN GLN PRO PRO VAL PRO TYR ARG GLN ILE
                    1013                                       1024
```

```
        16                                                        30
SEQRES: ASP ASP CYS PRO ALA LYS ALA ARG PRO GLN HIS ILE PHE TYR ARG
COORDS: ASP ASP CYS PRO ALA LYS ALA ARG PRO GLN HIS ILE PHE TYR ARG
        1025                                                    1039

        31                                                        45
SEQRES: ARG PHE LEU GLY LYS ASP GLY ARG ARG ASP PRO LYS CYS GLN TRP
COORDS: ARG PHE LEU GLY LYS ASP GLY ARG ARG ASP PRO LYS CYS GLN TRP
        1040                                                    1054

        46                                                        60
SEQRES: LYS PHE ALA VAL ILE PHE TRP GLY ASN ASP PRO TYR GLY LEU LYS
COORDS: LYS PHE ALA VAL ILE PHE TRP GLY ASN ASP PRO TYR GLY LEU LYS
        1055                                                    1069

        61                                                        75
SEQRES: LYS LEU SER GLN ALA PHE GLN PHE GLY GLY VAL LYS ALA GLY PRO
COORDS: LYS LEU SER GLN ALA PHE GLN PHE GLY GLY VAL LYS ALA GLY PRO
        1070                                                    1084

        76                                                        90
SEQRES: VAL SER CYS LEU PRO HIS PRO GLY PRO ASP GLN SER PRO ILE THR
COORDS: VAL SER CYS LEU PRO HIS PRO GLY PRO ASP GLN SER PRO ILE THR
        1085                                                    1099

        91                                                       105
SEQRES: TYR CYS VAL TYR VAL TYR CYS GLN ASN LYS ASP THR SER LYS LYS
COORDS: TYR CYS VAL TYR VAL TYR CYS GLN ASN LYS ASP THR SER LYS LYS
        1100                                                    1114

        106                                                      120
SEQRES: VAL GLN MET ALA ARG LEU ALA TRP GLU ALA SER HIS PRO LEU ALA
COORDS: VAL GLN MET ALA ARG LEU ALA TRP GLU ALA SER HIS PRO LEU ALA
        1115                                                    1129

        121                                                      135
SEQRES: GLY ASN LEU GLN SER SER ILE VAL LYS PHE LYS LYS PRO LEU PRO
COORDS: GLY ASN LEU GLN SER SER ILE VAL LYS PHE LYS LYS PRO LEU PRO
        1130                                                    1144

        136             140
SEQRES: LEU THR GLN PRO GLY
COORDS: LEU THR GLN   ?   ?
        1145    1147
```

PDB Chain_ID: D

```
        1                                                         15
SEQRES: SER HIS PRO ARG TYR GLN GLN PRO PRO VAL PRO TYR ARG GLN ILE
COORDS:   ?   ?   ? ARG TYR GLN GLN PRO PRO VAL PRO TYR ARG GLN ILE
                    1013                                        1024

        16                                                        30
SEQRES: ASP ASP CYS PRO ALA LYS ALA ARG PRO GLN HIS ILE PHE TYR ARG
COORDS: ASP ASP CYS PRO ALA LYS ALA ARG PRO GLN HIS ILE PHE TYR ARG
        1025                                                    1039

        31                                                        45
SEQRES: ARG PHE LEU GLY LYS ASP GLY ARG ARG ASP PRO LYS CYS GLN TRP
COORDS: ARG PHE LEU GLY LYS ASP GLY ARG ARG ASP PRO LYS CYS GLN TRP
        1040                                                    1054

        46                                                        60
SEQRES: LYS PHE ALA VAL ILE PHE TRP GLY ASN ASP PRO TYR GLY LEU LYS
COORDS: LYS PHE ALA VAL ILE PHE TRP GLY ASN ASP PRO TYR GLY LEU LYS
        1055                                                    1069

        61                                                        75
SEQRES: LYS LEU SER GLN ALA PHE GLN PHE GLY GLY VAL LYS ALA GLY PRO
COORDS: LYS LEU SER GLN ALA PHE GLN PHE GLY GLY VAL LYS ALA GLY PRO
        1070                                                    1084

        76                                                        90
SEQRES: VAL SER CYS LEU PRO HIS PRO GLY PRO ASP GLN SER PRO ILE THR
```

```
COORDS: VAL SER CYS LEU PRO HIS PRO GLY PRO ASP GLN SER PRO ILE THR
        1085                                                    1099

        91                                                       105
SEQRES: TYR CYS VAL TYR VAL TYR CYS GLN ASN LYS ASP THR SER LYS LYS
COORDS: TYR CYS VAL TYR VAL TYR CYS GLN ASN LYS ASP THR SER LYS LYS
        1100                                                    1114

        106                                                      120
SEQRES: VAL GLN MET ALA ARG LEU ALA TRP GLU ALA SER HIS PRO LEU ALA
COORDS: VAL GLN MET ALA ARG LEU ALA TRP GLU ALA SER HIS PRO LEU ALA
        1115                                                    1129

        121                                                      135
SEQRES: GLY ASN LEU GLN SER SER ILE VAL LYS PHE LYS LYS PRO LEU PRO
COORDS: GLY ASN LEU GLN SER SER ILE VAL LYS PHE LYS LYS PRO LEU PRO
        1130                                                    1144

        136             140
SEQRES: LEU THR GLN PRO GLY
COORDS: LEU THR GLN PRO   ?
        1145        1148


PDB Chain_ID: E

        1                                                         15
SEQRES: SER HIS PRO ARG TYR GLN GLN PRO PRO VAL PRO TYR ARG GLN ILE
COORDS:   ?   ? PRO ARG TYR GLN GLN PRO PRO VAL PRO TYR ARG GLN ILE
                1012                                            1024

        16                                                        30
SEQRES: ASP ASP CYS PRO ALA LYS ALA ARG PRO GLN HIS ILE PHE TYR ARG
COORDS: ASP ASP CYS PRO ALA LYS ALA ARG PRO GLN HIS ILE PHE TYR ARG
        1025                                                    1039

        31                                                        45
SEQRES: ARG PHE LEU GLY LYS ASP GLY ARG ARG ASP PRO LYS CYS GLN TRP
COORDS: ARG PHE LEU GLY LYS ASP GLY ARG ARG ASP PRO LYS CYS GLN TRP
        1040                                                    1054

        46                                                        60
SEQRES: LYS PHE ALA VAL ILE PHE TRP GLY ASN ASP PRO TYR GLY LEU LYS
COORDS: LYS PHE ALA VAL ILE PHE TRP GLY ASN ASP PRO TYR GLY LEU LYS
        1055                                                    1069

        61                                                        75
SEQRES: LYS LEU SER GLN ALA PHE GLN PHE GLY GLY VAL LYS ALA GLY PRO
COORDS: LYS LEU SER GLN ALA PHE GLN PHE GLY GLY VAL LYS ALA GLY PRO
        1070                                                    1084

        76                                                        90
SEQRES: VAL SER CYS LEU PRO HIS PRO GLY PRO ASP GLN SER PRO ILE THR
COORDS: VAL SER CYS LEU PRO HIS PRO GLY PRO ASP GLN SER PRO ILE THR
        1085                                                    1099

        91                                                       105
SEQRES: TYR CYS VAL TYR VAL TYR CYS GLN ASN LYS ASP THR SER LYS LYS
COORDS: TYR CYS VAL TYR VAL TYR CYS GLN ASN LYS ASP THR SER LYS LYS
        1100                                                    1114

        106                                                      120
SEQRES: VAL GLN MET ALA ARG LEU ALA TRP GLU ALA SER HIS PRO LEU ALA
COORDS: VAL GLN MET ALA ARG LEU ALA TRP GLU ALA SER HIS PRO LEU ALA
        1115                                                    1129

        121                                                      135
SEQRES: GLY ASN LEU GLN SER SER ILE VAL LYS PHE LYS LYS PRO LEU PRO
COORDS: GLY ASN LEU GLN SER SER ILE VAL LYS PHE LYS LYS PRO LEU PRO
        1130                                                    1144

        136             140
SEQRES: LEU THR GLN PRO GLY
COORDS: LEU THR GLN PRO   ?
        1145        1148
```

```
PDB Chain_ID: F

        1                                                         15
SEQRES: SER HIS PRO ARG TYR GLN GLN PRO PRO VAL PRO TYR ARG GLN ILE
COORDS:   ?   ?   ? ARG TYR GLN GLN PRO PRO VAL PRO TYR ARG GLN ILE
                    1013                                        1024

        16                                                        30
SEQRES: ASP ASP CYS PRO ALA LYS ALA ARG PRO GLN HIS ILE PHE TYR ARG
COORDS: ASP ASP CYS PRO ALA LYS ALA ARG PRO GLN HIS ILE PHE TYR ARG
        1025                                                    1039

        31                                                        45
SEQRES: ARG PHE LEU GLY LYS ASP GLY ARG ARG ASP PRO LYS CYS GLN TRP
COORDS: ARG PHE LEU GLY LYS ASP GLY ARG ARG ASP PRO LYS CYS GLN TRP
        1040                                                    1054

        46                                                        60
SEQRES: LYS PHE ALA VAL ILE PHE TRP GLY ASN ASP PRO TYR GLY LEU LYS
COORDS: LYS PHE ALA VAL ILE PHE TRP GLY ASN ASP PRO TYR GLY LEU LYS
        1055                                                    1069

        61                                                        75
SEQRES: LYS LEU SER GLN ALA PHE GLN PHE GLY GLY VAL LYS ALA GLY PRO
COORDS: LYS LEU SER GLN ALA PHE GLN PHE GLY GLY VAL LYS ALA GLY PRO
        1070                                                    1084

        76                                                        90
SEQRES: VAL SER CYS LEU PRO HIS PRO GLY PRO ASP GLN SER PRO ILE THR
COORDS: VAL SER CYS LEU PRO HIS PRO GLY PRO ASP GLN SER PRO ILE THR
        1085                                                    1099

        91                                                       105
SEQRES: TYR CYS VAL TYR VAL TYR CYS GLN ASN LYS ASP THR SER LYS LYS
COORDS: TYR CYS VAL TYR VAL TYR CYS GLN ASN LYS ASP THR SER LYS LYS
        1100                                                    1114

        106                                                      120
SEQRES: VAL GLN MET ALA ARG LEU ALA TRP GLU ALA SER HIS PRO LEU ALA
COORDS: VAL GLN MET ALA ARG LEU ALA TRP GLU ALA SER HIS PRO LEU ALA
        1115                                                    1129

        121                                                      135
SEQRES: GLY ASN LEU GLN SER SER ILE VAL LYS PHE LYS LYS PRO LEU PRO
COORDS: GLY ASN LEU GLN SER SER ILE VAL LYS PHE LYS LYS PRO LEU PRO
        1130                                                    1144

        136             140
SEQRES: LEU THR GLN PRO GLY
COORDS: LEU THR GLN PRO GLY
        1145            1149


PDB Chain_ID: G

        1                                                         15
SEQRES: SER HIS PRO ARG TYR GLN GLN PRO PRO VAL PRO TYR ARG GLN ILE
COORDS:   ?   ?   ? ARG TYR GLN GLN PRO PRO VAL PRO TYR ARG GLN ILE
                    1013                                        1024

        16                                                        30
SEQRES: ASP ASP CYS PRO ALA LYS ALA ARG PRO GLN HIS ILE PHE TYR ARG
COORDS: ASP ASP CYS PRO ALA LYS ALA ARG PRO GLN HIS ILE PHE TYR ARG
        1025                                                    1039

        31                                                        45
SEQRES: ARG PHE LEU GLY LYS ASP GLY ARG ARG ASP PRO LYS CYS GLN TRP
COORDS: ARG PHE LEU GLY LYS ASP GLY ARG ARG ASP PRO LYS CYS GLN TRP
        1040                                                    1054

        46                                                        60
SEQRES: LYS PHE ALA VAL ILE PHE TRP GLY ASN ASP PRO TYR GLY LEU LYS
COORDS: LYS PHE ALA VAL ILE PHE TRP GLY ASN ASP PRO TYR GLY LEU LYS
```

```
        1055                                              1069

        61                                                  75
SEQRES: LYS LEU SER GLN ALA PHE GLN PHE GLY GLY VAL LYS ALA GLY PRO
COORDS: LYS LEU SER GLN ALA PHE GLN PHE GLY GLY VAL LYS ALA GLY PRO
        1070                                              1084

        76                                                  90
SEQRES: VAL SER CYS LEU PRO HIS PRO GLY PRO ASP GLN SER PRO ILE THR
COORDS: VAL SER CYS LEU PRO HIS PRO GLY PRO ASP GLN SER PRO ILE THR
        1085                                              1099

        91                                                 105
SEQRES: TYR CYS VAL TYR VAL TYR CYS GLN ASN LYS ASP THR SER LYS LYS
COORDS: TYR CYS VAL TYR VAL TYR CYS GLN ASN LYS ASP THR SER LYS LYS
        1100                                              1114

        106                                                120
SEQRES: VAL GLN MET ALA ARG LEU ALA TRP GLU ALA SER HIS PRO LEU ALA
COORDS: VAL GLN MET ALA ARG LEU ALA TRP GLU ALA SER HIS PRO LEU ALA
        1115                                              1129

        121                                                135
SEQRES: GLY ASN LEU GLN SER SER ILE VAL LYS PHE LYS LYS PRO LEU PRO
COORDS: GLY ASN LEU GLN SER SER ILE VAL LYS PHE LYS LYS PRO LEU PRO
        1130                                              1144

        136             140
SEQRES: LEU THR GLN PRO GLY
COORDS: LEU THR GLN   ?   ?
        1145   1147


PDB Chain_ID: H

        1                                                   15
SEQRES: SER HIS PRO ARG TYR GLN GLN PRO PRO VAL PRO TYR ARG GLN ILE
COORDS:   ?   ?   ? ARG TYR GLN GLN PRO PRO VAL PRO TYR ARG GLN ILE
                    1013                                  1024

        16                                                  30
SEQRES: ASP ASP CYS PRO ALA LYS ALA ARG PRO GLN HIS ILE PHE TYR ARG
COORDS: ASP ASP CYS PRO ALA LYS ALA ARG PRO GLN HIS ILE PHE TYR ARG
        1025                                              1039

        31                                                  45
SEQRES: ARG PHE LEU GLY LYS ASP GLY ARG ARG ASP PRO LYS CYS GLN TRP
COORDS: ARG PHE LEU GLY LYS ASP GLY ARG ARG ASP PRO LYS CYS GLN TRP
        1040                                              1054

        46                                                  60
SEQRES: LYS PHE ALA VAL ILE PHE TRP GLY ASN ASP PRO TYR GLY LEU LYS
COORDS: LYS PHE ALA VAL ILE PHE TRP GLY ASN ASP PRO TYR GLY LEU LYS
        1055                                              1069

        61                                                  75
SEQRES: LYS LEU SER GLN ALA PHE GLN PHE GLY GLY VAL LYS ALA GLY PRO
COORDS: LYS LEU SER GLN ALA PHE GLN PHE GLY GLY VAL LYS ALA GLY PRO
        1070                                              1084

        76                                                  90
SEQRES: VAL SER CYS LEU PRO HIS PRO GLY PRO ASP GLN SER PRO ILE THR
COORDS: VAL SER CYS LEU PRO HIS PRO GLY PRO ASP GLN SER PRO ILE THR
        1085                                              1099

        91                                                 105
SEQRES: TYR CYS VAL TYR VAL TYR CYS GLN ASN LYS ASP THR SER LYS LYS
COORDS: TYR CYS VAL TYR VAL TYR CYS GLN ASN LYS ASP THR SER LYS LYS
        1100                                              1114

        106                                                120
SEQRES: VAL GLN MET ALA ARG LEU ALA TRP GLU ALA SER HIS PRO LEU ALA
COORDS: VAL GLN MET ALA ARG LEU ALA TRP GLU ALA SER HIS PRO LEU ALA
        1115                                              1129
```

```
        121                                                        135
SEQRES: GLY ASN LEU GLN SER SER ILE VAL LYS PHE LYS LYS PRO LEU PRO
COORDS: GLY ASN LEU GLN SER SER ILE VAL LYS PHE LYS LYS PRO LEU PRO
        1130                                                      1144

        136             140
SEQRES: LEU THR GLN PRO GLY
COORDS: LEU THR GLN   ?   ?
        1145   1147
```

PDB Chain_ID: I

```
        1                                                           15
SEQRES: SER HIS PRO ARG TYR GLN GLN PRO PRO VAL PRO TYR ARG GLN ILE
COORDS:   ?   ? PRO ARG TYR GLN GLN PRO PRO VAL PRO TYR ARG GLN ILE
                1012                                              1024

        16                                                          30
SEQRES: ASP ASP CYS PRO ALA LYS ALA ARG PRO GLN HIS ILE PHE TYR ARG
COORDS: ASP ASP CYS PRO ALA LYS ALA ARG PRO GLN HIS ILE PHE TYR ARG
        1025                                                      1039

        31                                                          45
SEQRES: ARG PHE LEU GLY LYS ASP GLY ARG ARG ASP PRO LYS CYS GLN TRP
COORDS: ARG PHE LEU GLY LYS ASP GLY ARG ARG ASP PRO LYS CYS GLN TRP
        1040                                                      1054

        46                                                          60
SEQRES: LYS PHE ALA VAL ILE PHE TRP GLY ASN ASP PRO TYR GLY LEU LYS
COORDS: LYS PHE ALA VAL ILE PHE TRP GLY ASN ASP PRO TYR GLY LEU LYS
        1055                                                      1069

        61                                                          75
SEQRES: LYS LEU SER GLN ALA PHE GLN PHE GLY GLY VAL LYS ALA GLY PRO
COORDS: LYS LEU SER GLN ALA PHE GLN PHE GLY GLY VAL LYS ALA GLY PRO
        1070                                                      1084

        76                                                          90
SEQRES: VAL SER CYS LEU PRO HIS PRO GLY PRO ASP GLN SER PRO ILE THR
COORDS: VAL SER CYS LEU PRO HIS PRO GLY PRO ASP GLN SER PRO ILE THR
        1085                                                      1099

        91                                                         105
SEQRES: TYR CYS VAL TYR VAL TYR CYS GLN ASN LYS ASP THR SER LYS LYS
COORDS: TYR CYS VAL TYR VAL TYR CYS GLN ASN LYS ASP THR SER LYS LYS
        1100                                                      1114

        106                                                        120
SEQRES: VAL GLN MET ALA ARG LEU ALA TRP GLU ALA SER HIS PRO LEU ALA
COORDS: VAL GLN MET ALA ARG LEU ALA TRP GLU ALA SER HIS PRO LEU ALA
        1115                                                      1129

        121                                                        135
SEQRES: GLY ASN LEU GLN SER SER ILE VAL LYS PHE LYS LYS PRO LEU PRO
COORDS: GLY ASN LEU GLN SER SER ILE VAL LYS PHE LYS LYS PRO LEU PRO
        1130                                                      1144

        136             140
SEQRES: LEU THR GLN PRO GLY
COORDS: LEU THR GLN PRO GLY
        1145           1149
```

PDB Chain_ID: J

```
        1                                                           15
SEQRES: SER HIS PRO ARG TYR GLN GLN PRO PRO VAL PRO TYR ARG GLN ILE
COORDS: SER HIS PRO ARG TYR GLN GLN PRO PRO VAL PRO TYR ARG GLN ILE
        1010                                                      1024

        16                                                          30
SEQRES: ASP ASP CYS PRO ALA LYS ALA ARG PRO GLN HIS ILE PHE TYR ARG
COORDS: ASP ASP CYS PRO ALA LYS ALA ARG PRO GLN HIS ILE PHE TYR ARG
        1025                                                      1039
```

```
        31                                                     45
SEQRES: ARG PHE LEU GLY LYS ASP GLY ARG ARG ASP PRO LYS CYS GLN TRP
COORDS: ARG PHE LEU GLY LYS ASP GLY ARG ARG ASP PRO LYS CYS GLN TRP
        1040                                                 1054

        46                                                     60
SEQRES: LYS PHE ALA VAL ILE PHE TRP GLY ASN ASP PRO TYR GLY LEU LYS
COORDS: LYS PHE ALA VAL ILE PHE TRP GLY ASN ASP PRO TYR GLY LEU LYS
        1055                                                 1069

        61                                                     75
SEQRES: LYS LEU SER GLN ALA PHE GLN PHE GLY GLY VAL LYS ALA GLY PRO
COORDS: LYS LEU SER GLN ALA PHE GLN PHE GLY GLY VAL LYS ALA GLY PRO
        1070                                                 1084

        76                                                     90
SEQRES: VAL SER CYS LEU PRO HIS PRO GLY PRO ASP GLN SER PRO ILE THR
COORDS: VAL SER CYS LEU PRO HIS PRO GLY PRO ASP GLN SER PRO ILE THR
        1085                                                 1099

        91                                                    105
SEQRES: TYR CYS VAL TYR VAL TYR CYS GLN ASN LYS ASP THR SER LYS LYS
COORDS: TYR CYS VAL TYR VAL TYR CYS GLN ASN LYS ASP THR SER LYS LYS
        1100                                                 1114

        106                                                   120
SEQRES: VAL GLN MET ALA ARG LEU ALA TRP GLU ALA SER HIS PRO LEU ALA
COORDS: VAL GLN MET ALA ARG LEU ALA TRP GLU ALA SER HIS PRO LEU ALA
        1115                                                 1129

        121                                                   135
SEQRES: GLY ASN LEU GLN SER SER ILE VAL LYS PHE LYS LYS PRO LEU PRO
COORDS: GLY ASN LEU GLN SER SER ILE VAL LYS PHE LYS LYS PRO LEU PRO
        1130                                                 1144

        136             140
SEQRES: LEU THR GLN PRO GLY
COORDS: LEU THR GLN   ?   ?
        1145   1147
```
